# Manganese Dioxide-Based pH-Responsive Multifunctional Nanoparticles Deliver Methotrexate for Targeted Rheumatoid Arthritis Treatment

**DOI:** 10.34133/bmr.0187

**Published:** 2025-05-14

**Authors:** Jingwen Jia, Min Liu, Han Yang, XiaoFang Li, Siyi Liu, Kexin Li, Jiulong Zhang, Xiuli Zhao

**Affiliations:** College of Pharmacy, Shenyang Pharmaceutical University, Shenyang, Liaoning 110016, China.

## Abstract

Rheumatoid arthritis (RA) is an autoimmune disease characterized by hypoxia and reactive oxygen species (ROS) overexpression, which cause inflammatory cascade and cartilage erosion. As representative inflammatory cells, macrophages produce many inflammatory factors, and intracellular ROS is abnormally elevated. Therefore, improving hypoxia and scavenging ROS are essential to inhibit the inflammatory response of synovial macrophages and cartilage destruction. Due to the complex microenvironment of RA and the single action of most anti-inflammatory and antioxidant drugs, as well as the difficulty in reversing the microenvironment with current formulations developed for ROS clearance, it is necessary to develop multifunctional nanoparticles (NPs) to achieve better therapeutic effects. In this work, we constructed a delivery system called PCM@MnO_2_ NPs, which could reduce inflammatory factors and improve the RA environment through multifunctional synergistic effects such as eliminating ROS and generating oxygen. Specifically, chondroitin sulfate was used to form NPs with methotrexate (MTX) through electrostatic interactions and hydrogen bonding and further loaded with MnO_2_ to form CM@MnO_2_ NPs. Furthermore, modification of polydopamine on the surface of CM@MnO_2_ NPs improved the stability of the formulation and extended the cycle time. Under the acidic (pH 6.5) microenvironment of RA, polydopamine shells were dissociated. Chondroitin sulfate could target inflammatory macrophages via the CD44 receptor and subsequently release MTX and MnO_2_ under low-intracellular-pH (pH 5.2) conditions. MnO_2_ could decompose and consume ROS and further produce oxygen in the process of decomposing H_2_O_2_, alleviating the hypoxic microenvironment of RA. In addition, MTX could also inhibit the secretion of cytokines. Overall, by regulating the RA microenvironment through the various synergistic effects mentioned above, it could promote macrophage polarization and alleviate RA progression. The experimental results in vitro and in vivo indicated that pH-responsive PCM@MnO_2_ NPs could accumulate in inflammatory joints by the extravasation through leaky vasculature and subsequent inflammatory cell-mediated sequestration (ELVIS) effect, enhance the precise delivery of MTX by targeting RA macrophages, and effectively alleviate the progression of disease and reduce the symptoms of collagen-induced arthritis mouse models. In general, using multifunctional synergistic therapy for RA is an effective potential strategy.

## Introduction

Rheumatoid arthritis (RA) is a chronic autoimmune joint disorder characterized by synovial inflammation and cartilage matrix degradation, often accompanied by synovial hyperplasia and oxidative stress [1,2]. Glucocorticoids, nonsteroidal anti-inflammatory drugs, and disease-modifying antirheumatic drugs all are frequently employed in clinical practice to control and delay RA progression [3]. Methotrexate (MTX), being a first-line antirheumatic drug for RA, is employed to inhibit the generation of pro-inflammatory cytokines. Unfortunately, MTX possesses poor solubility in water, low bioavailability, and severe toxicity due to weak targeting of inflammatory cells including macrophages [4,5]. At present, nanotargeting systems are designed to encapsulate MTX and deliver it to RA lesions to improve the therapeutic effect and decrease toxic side effects [6]. However, nanopreparations still have problems such as difficult degradation and high toxicity. Chondroitin sulfate (CS), as a negatively charged naturally sulfated glycosaminoglycan, can be self-assembled into nanocarriers due to its excellent water solubility [7,8]. Moreover, CS possess an excellent affinity for CD44 receptors markedly increased on the surface of inflammatory macrophages, and studies have shown that CS and MTX could be combined through electrostatic and hydrogen bonding [9]. Therefore, CS with good biocompatibility was used as the main component for constructing RA drug nanocarriers to encapsulate MTX [10,11].

Numerous clinical studies have reported that the RA mechanism was associated with oxidative stress in the presence of excessive production of reactive oxygen species (ROS) [12]. Excessive ROS increases the level of pro-inflammatory cytokines in inflammatory sites [13]; recruits and activates macrophages into the M1 phenotype [14], which will promote the inflammatory response by secreting pro-inflammatory cytokines; and recruits other immune cells to promote the progression of RA [15,16]. In addition, the increased oxygen requirement of cells during inflammation of an RA joint induces a hypoxic microenvironment as synovial tissue proliferation exceeds angiogenesis [17,18]. The overexpression of hypoxia-inducible factor 1α (HIF-1α) in RA affects the balance of polarization from M1 to M2 phenotype. Multiple factors interact in RA to form a vicious circle, leading to increasing inflammation [14,19]. Therefore, removing excess ROS from joints, improving hypoxia, and modulating the microenvironment at the site of inflammation are essential to alleviate RA progression. Studies have shown that compatible catalyzed nanoparticles (NPs), such as MnO_2_, CeO_2_, and CaO_2_ NPs [20,21], produce oxygen through a self-sufficient process with the ability to decompose peroxidases and produce oxygen in an anoxic environment rich in H_2_O_2_ [22]. Under the acidic pH condition of RA, MnO_2_ can not only consume abnormally elevated H_2_O_2_ but also alleviate the hypoxia of the microenvironment by continuously producing oxygen, thereby regulating the inflammatory microenvironment of RA and promoting the phenotypic repolarization of macrophages [18,23,24]. Unlike many nonbiodegradable inorganic nanomaterials, MnO_2_ NPs are broken down into harmless, water-soluble Mn^2+^ and quickly excreted through the kidneys, so there is no long-term toxicity problem [25]. However, MnO_2_ NPs do not have colloidal stability in biological fluids such as synovial fluids [22]. Encapsulating MnO_2_ in CS–MTX could effectively mitigate the above drawbacks. In addition, the catechol structure in dopamine (DA) generates strong electrostatic repulsion due to the negative charge on the NPs [26], which improves the stability of polydopamine (PDA)-coated NPs (CM@MnO_2_ NPs). It is also widely used in inflammation because of its abundant reducing groups, which have strong antioxidant activity and efficient ROS scavenging efficiency [19,27,28].

**Fig. 1.**
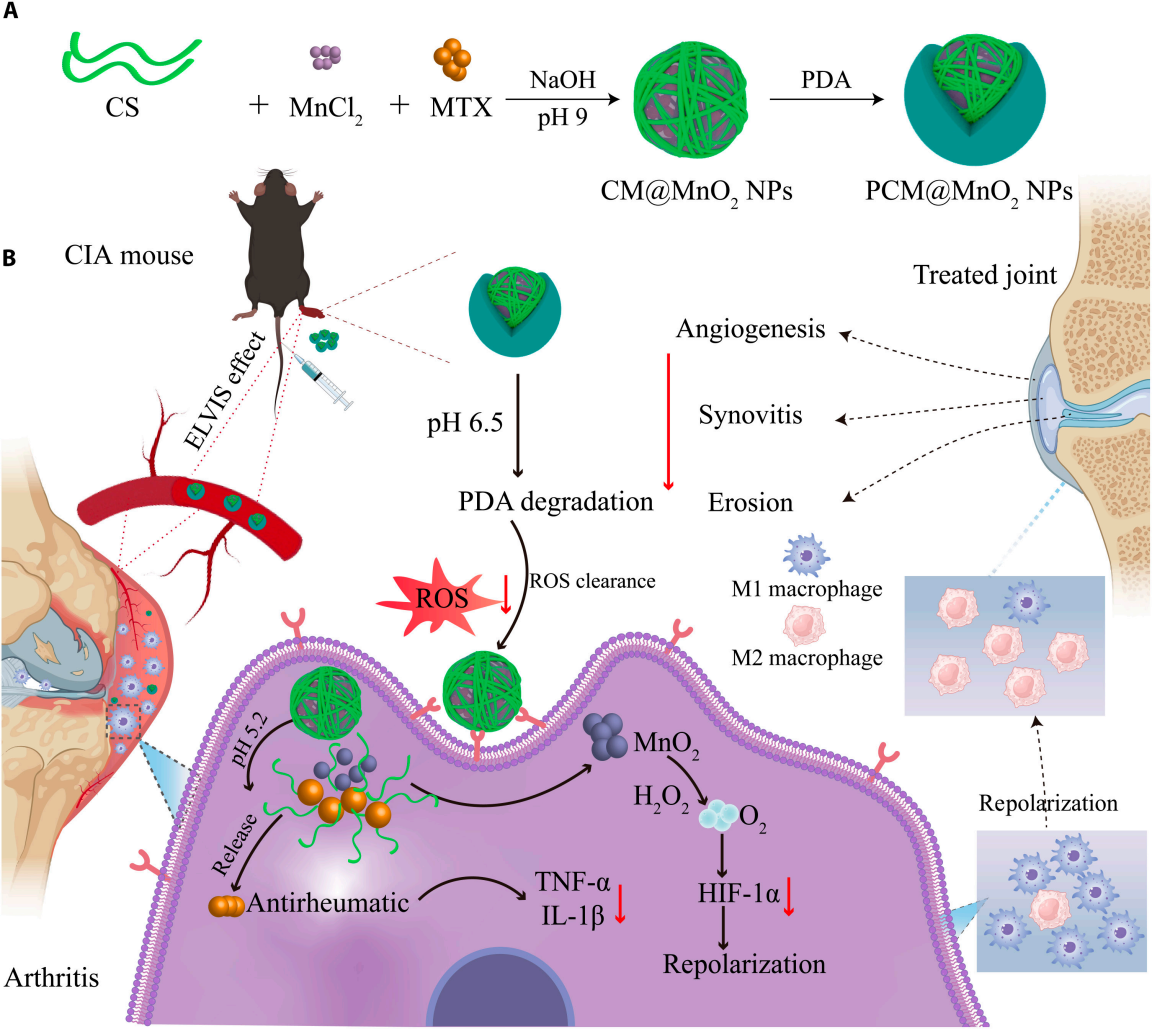
Construction and application for rheumatoid arthritis (RA) therapy of PCM@MnO_2_ nanoparticles (NPs). (A) PCM@MnO_2_ NPs were connected by chondroitin sulfate (CS) and methotrexate (MTX) through electrostatic interaction and hydrogen bonding, and the pH was adjusted to encapsulate MnO_2_ and form a polydopamine (PDA) shell. (B) Application of PCM@MnO_2_ NPs in RA treatment and related mechanisms. Under RA acidic conditions, PDA was degraded from PCM@MnO_2_ NPs to scavenge reactive oxygen species (ROS). CM@MnO_2_ NPs were internalized by macrophages through interaction between CS and CD44. Under intracellular conditions of low pH, CM@MnO_2_ NPs disintegrated to release MnO_2_ and MTX, which could produce O_2_ and inhibit inflammatory factors, respectively. PCM@MnO_2_ NPs effectively treated RA by improving the oxidative stress microenvironment. CIA, collagen-induced arthritis; ELVIS, extravasation through leaky vasculature and subsequent inflammatory cell-mediated sequestration; TNF-α, tumor necrosis factor-alpha; IL-1β, interleukin 1 beta; HIF-1α, hypoxia-inducible factor 1α.

In the current work, multifunctional NPs (PCM@MnO_2_ NPs) hybridized with PDA and MnO_2_ were synthesized simply and efficiently with the help of DA under alkaline conditions by small-molecule oxidative polymerization (Fig. 1). PDA coating could increase the stability of NPs and blood circulation time. After the accumulation of PCM@MnO_2_ NPs in inflamed joints, PDA disintegrated, and CM@MnO_2_ NPs were taken up by macrophages via CD44 receptors, thereby achieving precise delivery of MTX. MnO_2_ reacted with H_2_O_2_ to produce O_2_, alleviated the hypoxic microenvironment of RA, and induced M1–M2 repolarization. PCM@MnO_2_ NPs could enhance efficacy and reduce toxicity through targeted delivery of MTX and could reduce inflammation more efficiently with a multifunctional synergistic nanotherapy for RA.

## Materials and Methods

### Materials

CS, MTX, DA, manganese chloride tetrahydrate (MnCl_2_·4H_2_O), 2′,7′-dichlorodihydrofluorescein diacetate (DCFH-DA), and lipopolysaccharide (LPS) were purchased from Sigma Technology Co., Ltd (USA). Chick type II collagen and complete Freund’s adjuvant (CFA) were provided by Chondrex Inc. (USA). 2,2-Diphenyl-2-picrylhydrazyl (DPPH•) was obtained from Yuanye Biotechnology (Shanghai, China). The detection kits for oxidative-stress-related indicators were purchased from Jiancheng Bioengineering Institute (Nanjing, China). RPMI-1640 cell culture medium and Cell Counting Kit-8 (CCK-8) assay kit were provided by Meilun Biotechnology Co., Ltd. (Dalian, China). Enzyme-linked immunosorbent assay (ELISA) kits including mouse aspartate aminotransferase (AST), alanine aminotransferase (ALT), blood urea nitrogen (BUN), and creatinine (Cre); mouse tumor necrosis factor-alpha (TNF-α); and mouse IL-1β (interleukin 1 beta) were obtained from Elabscience Biotechnology Co., Ltd. (Wuhan, China).

### Animals and cell culture

Male C57BL/6 mice (9 to 12 weeks, 18 to 24 g) were obtained from the Animal Center of Shenyang Pharmaceutical University. Relevant experiments were performed according to the guidelines of the Animal Ethics Committee.

RAW264.7 cells were derived from the Shanghai Cell Bank of the Chinese Academy of Sciences, and chondrocytes were obtained from the articular cartilage of C57BL/6 mice afflicted with collagen-induced arthritis (CIA) and cultured in RPMI-1640 (containing 10% fetal bovine serum [FBS]) at 37 °C and 5% CO_2_.

### Preparation and characterization of PCM@MnO_2_ NPs

One milliliter of MnCl_2_ (5 mg/ml) was added to 1 ml of CS (10 mg/ml) and stirred for 30 min, and then 1 ml of MTX (5 mg/ml) was added. The pH was adjusted to 9.0 using 0.1 M NaOH after 30 min. The stirring of the solution was continued for 4 h; CS–MTX-MnO_2_ NPs (termed CM@MnO_2_ NPs) were obtained by centrifugation at 13,000 rpm for 10 min to remove free MTX. Subsequently, PDA–CS–MTX–MnO_2_ NPs (termed PCM@MnO_2_ NPs) were prepared by coating with PDA, which was obtained by self-polymerization of DA in an alkaline environment [29,30]. Briefly, 1 ml of DA (4 mg/ml) was added to CM@MnO_2_ NP solution and purified by centrifugation at 13,000 rpm for 10 min after stirring for 30 min. Similarly, CS–MTX NPs (termed CM NPs) were prepared through the above method without MnO_2_. Specifically, 1 ml of MTX (5 mg/ml) was added to 1 ml of CS (10 mg/ml) and stirred for 2 to 3 h at 700 rpm to obtain CM NPs. In addition, since CM NPs were unstable under high-speed centrifugation, a dialysis membrane (molecular weight cutoff [MWCO]: 3.5 kDa) was used to remove unencapsulated CS and MTX. An ultraviolet–visible (UV–vis) spectrophotometer was employed to determine the characteristic absorption peaks of MTX, CM NPs, CM@MnO_2_ NPs, and PCM@MnO_2_ NPs. In addition, the characteristic absorption peaks of CS and CM NPs were measured by a Fourier transform infrared spectrophotometer, and x-ray photoelectron spectroscopy was employed to analyze the chemical composition of CM@MnO_2_ NPs.

The dynamic light scattering and electrophoretic light scattering techniques were used to determine the particle size, polydispersity index (PDI), and zeta potential of the sample. The morphology of NPs was further observed by transmission electron microscopy (JEM-2100, Japan).

### Encapsulation efficiency (EE%)

The CM NP suspension was dialyzed for 10 h with a dialysis membrane (MWCO: 3.5 kDa). CM@MnO_2_ NPs and PCM@MnO_2_ NPs were centrifuged for 10 min at 13,000 rpm, and supernatants were taken; 200 μl of the filtrate was separately added to 800 μl of dimethyl sulfoxide. The absorbance of the mixture was determined at 305 nm using a UV-1800 spectrophotometer. The encapsulation efficiency of MTX was calculated according to the following formula:

$$\mathrm{EE}\%=\frac{Q_{t}-Q_{s}}{Q_{t}}\times 100\%,\;\mathrm{EE}\%=\frac{Q_{c}}{Q_{t}}\times 100\%$$ (1)

*Q_t_*, *Q_s_*, and *Q_c_* refer to the total amount, the amount of the supernatant, and the amount of MTX after dialysis, respectively.

### Stability of PCM@MnO_2_ NPs

CM NPs, CM@MnO_2_ NPs, and PCM@MnO_2_ NPs were additionally added to water, phosphate-buffered saline (PBS), or PBS (including 10% FBS, pH 7.4) and incubated for 4 d at 4 °C to assess stability. Briefly, 1 ml of CM NPs, CM@MnO_2_ NPs, and PCM@MnO_2_ NPs was respectively dispersed in 4 ml of different media. The particle size, PDI, and zeta potential of the CM NPs, CM@MnO_2_ NPs, and PCM@MnO_2_ NPs were measured daily.

### Drug release of MTX in vitro

The release of MTX in CM NPs, CM@MnO_2_ NPs, and PCM@MnO_2_ NPs was performed by dialysis. Simply, 2 ml of CM@MnO_2_ NPs or PCM@MnO_2_ NPs was added to the dialysis membrane (MWCO: 3.5 kDa) and immersed in 40 ml of PBS of different pHs (7.4, 6.5, and 5.2) under continuous agitation at 37 °C and 100 rpm. At specific time points (0, 0.5, 1, 2, 4, 6, 8, 12, 24, and 48 h), 2 ml of the release solution was taken for testing and PBS with equal temperature and volume was added. UV–vis spectroscopy was used to measure the concentration of MTX.

### CM@MnO_2_ NPs’ and PCM@MnO_2_ NPs’ H_2_O_2_ decomposition and in vitro oxygen generation

To assess the pH response of PCM@MnO_2_ NPs, the determination of terephthalic acid (TA) was carried out. TA will exhibit fluorescence at 360 nm after adding H_2_O_2_. PCM@MnO_2_ NPs (100 μg/ml) were mixed with TA and H_2_O_2_ (1 mM), and the fluorescence intensities of TA at different pHs (7.4, 6.5, and 5.2) were detected at 310/420-nm wavelengths using a multiplate reader.

The hydrogen peroxide scavenging capacity was evaluated by measuring TA. Specifically, CM@MnO_2_ NPs or PCM@MnO_2_ NPs with different concentrations (0, 50, 100, 200, and 500 μg/ml) were mixed with TA and H_2_O_2_ (1 mM), respectively.

CM NPs, CM@MnO_2_ NPs, and PCM@MnO_2_ NPs (100 μg/ml) were added to PBS containing 1 mM H_2_O_2_ and stirred continuously. The amount of oxygen produced per minute by different NPs was measured by a dissolved oxygen meter.

### ROS scavenging ability of PCM@MnO_2_ NPs

#### DPPH scavenging

PCM@MnO_2_ NPs with various concentrations (50, 100, 200, and 500 μg/ml) were added to DPPH ethanol solution (0.1 mM) and reacted against light for 30 min. The fluorescence intensity of the remaining DPPH· was detected at 517 nm within 30 min (at 2 time points of 5 and 30 min). The absorbance within the UV–vis spectrum in the range of 450 to 650 nm was also recorded. The clearance of PCM@MnO_2_ NPs was calculated according to the clearance formula:

$$\text{Clearance}=\left(1-\frac{A_{\text{sample}}-A_{\text{blank}}}{A_{\text{control}}}\right)\times 100\%$$ (2)

#### H_2_O_2_ scavenging

The H_2_O_2_ scavenging capacity was assessed by measuring the H_2_O_2_ content. PCM@MnO_2_ NPs with different concentrations (50, 100, 200, and 500 μg/ml) were incubated with H_2_O_2_ (400 μM). After incubation at 37 °C for 40 min, titanium sulfate was added to detect the remaining H_2_O_2_. Finally, UV–vis absorbance in the range of 385 to 435 nm was recorded with a microplate reader.

#### O_2_^−^ scavenging

O_2_^−^ was produced by xanthine and xanthine oxidase. To assess O_2_^−^ clearance capacity, PCM@MnO_2_ NPs with different concentrations (50, 100, 200, and 500 μg/ml) were added and incubated at 37 °C for 40 min. The Griess reagent was added for chromogenic reaction, and the absorbance within the UV–vis spectrum in the range of 450 to 650 nm was recorded.

#### OH scavenging

In the Mn^2+^/H_2_O_2_ system, ·OH was generated through a Fenton-like reaction. PCM@MnO_2_ NPs at different concentrations (50, 100, 200, and 500 μg/ml) were added to evaluate the ·OH scavenging capacity. After incubation at 37 °C for 30 min, the Griess reagent was added to generate the red substance for further chromogenic reaction, and the microplate reader was employed to record the UV–vis spectral absorbance within 400 to 800 nm.

#### NO scavenging

The nitrate concentration was quantified using a modified Griess assay, where nitrate reductase converted NO_3_^−^ to NO_2_^−^, producing a measurable color change. PCM@MnO_2_ NPs with different concentrations (50, 100, 200, and 500 μg/ml) were added. By incubation and centrifugation, the supernatant was taken and the chromogenic agent was added for color development, and the absorbance within the UV–vis spectrum within 400 to 650 nm was measured by microplate reader.

### Hemolysis test

Red blood cells were obtained by centrifuging (1,500 rpm, 15 min) and washing fresh mouse blood with PBS. Different concentrations (50 to 400 μg/ml) of CM NPs, CM@MnO_2_ NPs, and PCM@MnO_2_ NPs were incubated with erythrocyte solutions (2%) for 4 h and were centrifuged to obtain the supernatant (3,000 rpm, 5 min). The red blood cell suspensions treated with H_2_O and PBS were selected as the positive and negative controls, respectively. The absorbance at 540 nm was measured to calculate the hemolysis percentage with a microplate reader. According to the hemolysis rate formula,

$$\text{Hemolysis}\%=\frac{OD_{\text{sample}}-OD_{\text{negative}}}{OD_{\text{positive}}-OD_{\text{negative}}}\times 100\%$$ (3)

### Cytotoxicity assay

The cytotoxicity of MTX, CM NPs, CM@MnO_2_ NPs, and PCM@MnO_2_ NPs was investigated by quantitative CCK-8 cell viability. RAW264.7 cells were initially seeded into 96-well plates (1 × 10^4^ cells/well) and incubated for 24 h with or without LPS (10 μg/ml). Then, different concentrations (10, 20, 50, 100, and 200 μg/ml) of MTX, CM NPs, CM@MnO_2_ NPs, and PCM@MnO_2_ NPs were added successively and incubated for 24 h. After that, 10 μl of CCK-8 was added and reacted for 4 h. A microplate reader was used to determine the absorbance at 450 nm. Cell viability was obtained by the following formula:

$$\text{Cell viability}=\frac{A_{s}-A_{b}}{A_{c}-A_{b}}\times 100\%$$ (4)

*A_s_*, *A_c_*, and *A_b_* represent the absorbance of the experimental wells, the control wells, and the blank wells, respectively, where the experimental wells contained cells, CCK-8, and the preparation; the mediators in the control wells were cells and CCK-8; and the blank wells contained only CCK-8.

### In vitro cell uptake

RAW264.7 cells were initially seeded into 12-well plates (1.0 × 10^5^ cells/well) and incubated for 24 h with or without LPS (10 μg/ml). After that, coumarin-6-loaded (5 μg/ml) NPs (CM NPs, CM@MnO_2_ NPs, and PCM@MnO_2_ NPs) were added to pristine and activated RAW264.7 cells, respectively, and incubated for 4 h. Subsequently, after the cells were washed with PBS for 3 times, 4% paraformaldehyde and 4′,6-diamidino-2-phenylindole (1 μg/ml) were added sequentially for cell fixation (20 min) and nucleus staining (10 min). Finally, the cellular uptake was visualized by confocal laser scanning microscopy (CLSM). RAW264.7 cells were reinoculated into 12-well plates (1.0 × 10^5^ cells/well) and incubated for 24 h with or without LPS (10 μg/ml) for quantitative analysis. Then, coumarin-6-loaded (5 μg/ml) CM NPs, CM@MnO_2_ NPs, and PCM@MnO_2_ NPs were added and incubated for 4 h. In addition, cells were washed 3 times with PBS and collected to determine the average fluorescence intensity of CM NPs, CM@MnO_2_ NPs, and PCM@MnO_2_ NPs by flow cytometry (FCM).

### ROS scavenging activity and determination of the expression level of cytokines

The ROS clearance ability of PCM@MnO_2_ NPs was detected by CLSM and FCM. After RAW264.7 cells were seeded into 12-well plates (1.0 × 10^5^ cells/well), they were then incubated with or without LPS (10 μg/ml) for 24 h. After incubation with CM NPs, CM@MnO_2_ NPs, and PCM@MnO_2_ NPs for 24 h, DCFH-DA (20 μM) was incubated with cells at 37 °C for 45 min and the cells were further imaged by CLSM. In addition, for the collected cells, the fluorescence intensity of intracellular ROS was further measured by FCM.

### Determination of the expression level of cytokines

The protein expression level of TNF-α and matrix metalloproteinase-2 (MMP2) was determined by western blot. After RAW264.7 cells were seeded into 6-well plates, LPS (10 μg/ml) was added and incubated for 24 h. Subsequently, the cells were treated with CM NPs, CM@MnO_2_ NPs, and PCM@MnO_2_ NPs for 4 h. The total protein concentration was extracted and quantified using a bicinchoninic acid kit. Proteins were then denatured (95 °C, 5 min), run on sodium dodecyl sulfate–polyacrylamide gel electrophoresis, and transferred to polyvinylidene fluoride membranes (0.22 μm). Afterward, the membranes were blocked (5% bovine serum albumin, 2 h) and incubated with the corresponding antibody (against β-actin, TNF-α, or MMP2) overnight at 4 °C, followed by incubation with secondary antibodies (horseradish peroxidase conjugated) at room temperature for 1 h. Finally, the bands were imaged using a Tanon Imager 1600 system. In addition, an ELISA kit was used to quantify the levels of TNF-α and IL-1β. RAW264.7 cells were first seeded into 96-well plates (1 × 10^5^ cells/well) and activated with LPS (10 μg/ml). Then, the cells were treated with CM NPs, CM@MnO_2_ NPs, and PCM@MnO_2_ NPs for 4 h. Lastly, the concentrations of TNF-α and IL-1β were detected using an ELISA kit. To further verify the chondroprotective effect of PCM@MnO_2_ NPs, chondrocytes were seeded into 96-well plates (1 × 10^4^ cells/well) and incubated for 24 h with IL-1β (100 ng/ml), and the various concentrations (10, 20, 50, 100, and 200 μg/ml) of CS, MTX, CM NPs, CM@MnO_2_ NPs, and PCM@MnO_2_ NPs were added and incubated for 24 h. Finally, the CCK-8 assay was performed to investigate the effect of NPs on chondrocyte proliferation.

### Repolarization activity of PCM@MnO_2_ NPs

For immunofluorescence assay, RAW264.7 cells were seeded into 12-well plates (1.0 × 10^5^ cells/well) and incubated for 24 h. After treating with LPS (10 μg/ml), PCM@MnO_2_ NPs were added to each well incubation for 24 h. Subsequently, the cells were fixed with 4% paraformaldehyde and incubated with inducible nitric oxide synthase (iNOS), CD206, or CD68 primary antibodies overnight at 4 °C. After staining with fluorescence-labeled secondary antibodies for 1 h, cells were imaged by fluorescence microscopy.

### Establishment of the CIA model

The C57BL/6 CIA mouse model was established with reference to the literature [31]. Mice were injected with 100 μg of an emulsion formed from chicken type II collagen and CFA collagen to establish primary immunity. First, chicken type II collagen was diluted to 4 mg/ml using 0.1 M ice acetic acid and incubated overnight on a shaker at 4 °C. Then, an equal volume of CFA was placed in a mortar on ice, and the collagen solution was added dropwise while being stirred until droplets that aggregate without dispersing were formed, indicating that emulsification was complete. Finally, 0.1 ml of the collagen emulsion was injected subcutaneously into the mouse at the base of the tail, approximately 1 cm anterior to the base, and into the right hind ankle to elicit an immune response. After 21 d, to trigger a second immunity, the mice were again injected subcutaneously with the same amount of the emulsion. The joints of the mice were observed and showed significant swelling. The mouse model was successfully established after 7 d of secondary immunization. Arthritis of modeled mice can be scored according to the degree of erythema and swelling. The score ranges from 0 to 4. (0: no swelling; 1: ankle redness and mild swelling; 2: redness and mild swelling spreading to the midfoot; 3: redness and moderate swelling at the joint; 4: severe redness and swelling at the joint). In each experimental group, all mice scored above 3, indicating a high success rate of model establishment.

### In vivo targeting evaluation

To evaluate the targeting ability of each NP on mice, the CIA mice were administered with 1,1′-dioctadecyl-3,3,3′,3′-tetramethylindotricarbocyanine iodide (DiR)-loaded CM NPs, CM@MnO_2_ NPs, and PCM@MnO_2_ at an equivalent dose of MTX of 5 mg/kg. Subsequently, mice were imaged with the FX Pro imaging system (Bruker, Inc., USA; excitation/emission: 720 nm/790 nm) at 1, 2, 4, and 8 h. Moreover, the spleen, heart, lungs, kidneys, and liver were collected and imaged with the FX Pro imaging system.

### In vivo therapeutic efficacy

After CIA mice were randomly divided into 5 groups (*n* = 6), they were injected intravenously with normal saline, MTX, CM NPs, CM@MnO_2_ NPs, and PCM@MnO_2_ NPs (MTX 5 mg/kg), respectively. Healthy mice represented the control group. CIA mice were treated intravenously with saline, MTX, CM NPs, CM@MnO_2_ NPs, and PCM@MnO_2_ NPs every 3 d. Meanwhile, the paw thickness, ankle diameter, and arthritis scores of CIA mice were recorded every 3 d during the experiment. Five days after the last administration, CIA mice were sacrificed and ankle specimens were collected. The joints were stained with hematoxylin–eosin (H&E) and Safranin O–fast green (SO-FG) to observe the articular cartilage injury of mice. The samples were initially immersed in 4% paraformaldehyde solution and embedded in paraffin, followed by H&E and SO-FG staining, and ultimately observed under a microscope.

Moreover, immunofluorescence staining was used to study the changes in macrophages in RA joints. After preprocessing, the sections were stained overnight with iNOS or CD206 antibodies at 4 °C and added with fluorescently labeled secondary antibodies, followed by incubation for 1 h. Finally, the sections were observed by fluorescent microscopy.

### Immunofluorescence and inflammatory factors

The inhibitory effect of each preparation was evaluated by observing the expression of TNF-α, IL-1β, and HIF-1α in the inflammatory joints after the end of treatment. Briefly, after a series of procedures such as decalcification and antigen recovery, joint sections were first stained for TNF-α, IL-1β, and HIF-1α antibodies overnight at 4 °C, followed by the addition of secondary antibodies and incubation for 1 h. The sections were observed using a microscope and the concentration of TNF-α and IL-1β in the serum of mice was quantified by an ELISA kit. Briefly, the diluted serum samples were added to a microtiter plate and incubated, followed by the addition of enzyme-linked secondary antibodies, and the substrate was added for color development and the absorbance was measured to analyze the levels of TNF-α and IL-1β.

### In vivo safety evaluation

The body weight of CIA mice was continuously recorded during administration. On day 5 after the last treatment, blood was collected and serum was obtained by centrifugation. An ELISA kit was employed to detect the levels of AST, ALT, BUN, and Cre to evaluate the safety of the preparation. The collected heart, liver, spleen, lung, and kidneys were fixed with 4% paraformaldehyde and stained with H&E.

### Statistical analysis

The quantitative results were expressed as mean ± standard deviation (SD). GraphPad Prism 10 (GraphPad Software, CA, USA) was used for other statistical analyses. In addition, differences between 2 groups and between multiple groups were evaluated using the Student *t* test and analysis of variance (ANOVA). *P < 0.05, **P < 0.01, ***P < 0.001, and ****P < 0.0001 indicated statistical significance.

## Results and Discussion

### Characterization of PCM@MnO_2_ NPs

CS was mixed with MTX to prepare CM NPs. In an alkaline dimethyl sulfoxide/distilled water medium, the deprotonation of –SO_3_H and –COOH in CS leads to the connection of MTX through electrostatic attraction and hydrogen bonding [9]. MnO_2_ was dispersed in the NP matrix (CM@MnO_2_ NPs) by adjusting the pH to 9 with NaOH. Previous laboratory studies have shown that PDA is an effective surface modification strategy to improve stability, enhance protein affinity, and prolong cycle time [32,33]. Therefore, coating the CM@MnO_2_ NPs with PDA (PCM@MnO_2_ NPs) could prevent drug leakage and further improve the stability of the NPs.

The characteristic peak of MTX was observed at 1,552 cm^−1^, and the movement of 1,251 cm^−1^ (S=O stretch) to 1,307 cm^−1^ in CS–MTX indicated an electrostatic interaction between CS and MTX. The –OH band in CS moved from 3,467 to 3,352 cm^−1^ in CS–MTX, which was related to the hydrogen bond between CS and MTX. The 1,421 cm^−1^ C–N band of CS moved to 1,446 cm^−1^ of CS–MTX (Fig. S1). The above results showed the successful connection between CS and MTX.

**Fig. 2.**
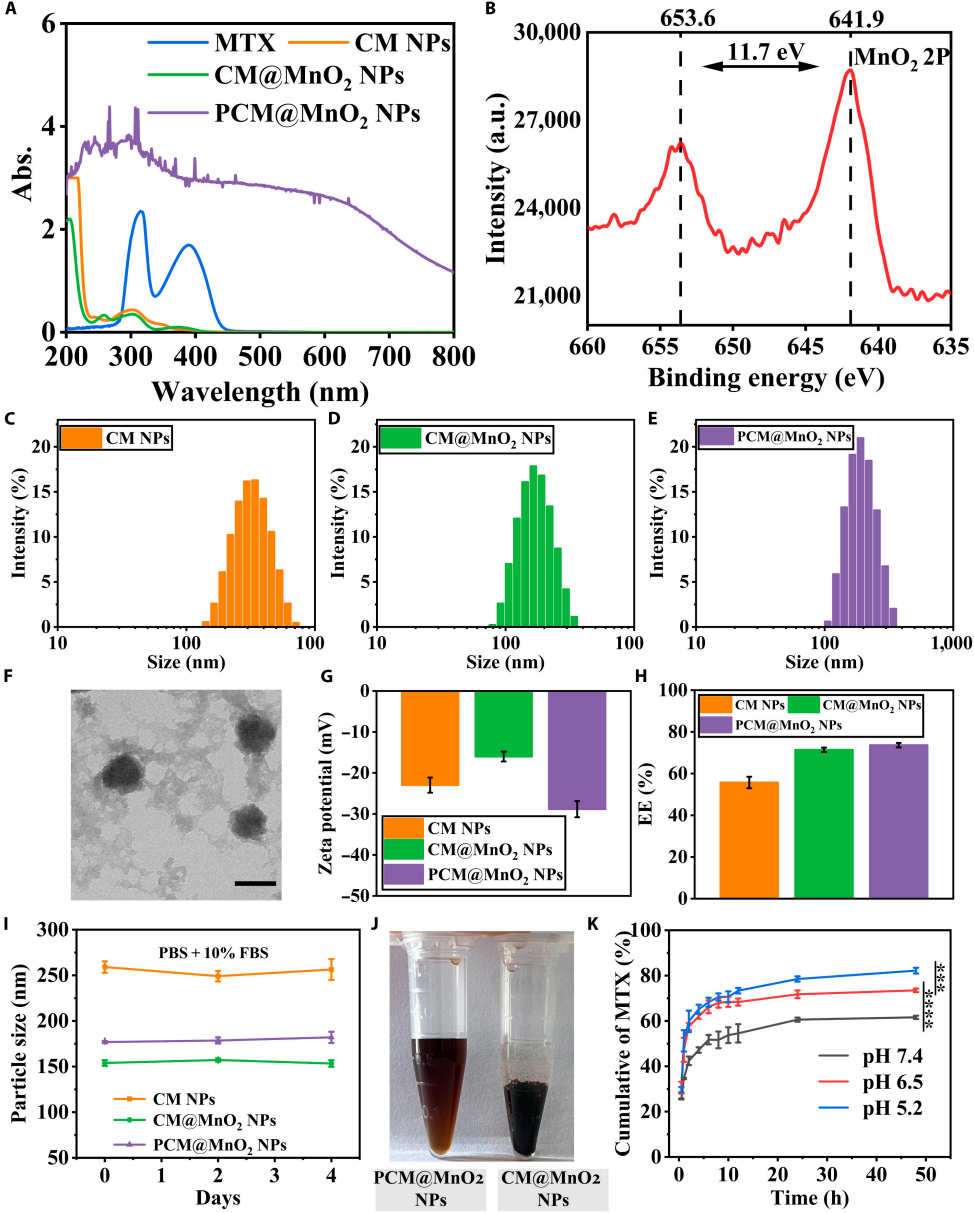
Characterization of CM NPs, CM@MnO_2_ NPs, and PCM@MnO_2_ NPs. (A) Ultraviolet–visible (UV–vis) spectra. (B) X-ray photoelectron spectroscopy (XPS) spectrum of CM@MnO_2_ NPs. Particle sizes of (C) CM NPs, (D) CM@MnO_2_ NPs, and (E) PCM@MnO_2_ NPs. (F) Transmission electron microscopy (TEM) image of PCM@MnO_2_ NPs; scale bar = 200 nm. (G) Zeta potential diagram of CM NPs, CM@MnO_2_ NPs, and PCM@MnO_2_ NPs. (H) Encapsulation efficiency (EE%) of MTX in CM NPs, CM@MnO_2_ NPs, and PCM@MnO_2_ NPs. (I) The serum stability of CM NPs, CM@MnO_2_ NPs, and PCM@MnO_2_ NPs (*n* = 3). (J) The photos of CM@MnO_2_ NPs and PCM@MnO_2_ NPs scattered in phosphate-buffered saline (PBS). (K) In vitro release of MTX from PCM@MnO_2_ NPs at different pHs (*n* = 3). Data were analyzed by one-way analysis of variance (ANOVA) (***P < 0.001; ****P < 0.0001). FBS, fetal bovine serum.

CM NPs and CM@MnO_2_ NPs exhibited the characteristic absorption peak of MTX (characteristic absorption peak of MTX at 305 nm [19]). The absorption peak of PCM@MnO_2_ NPs was observed at 280 nm, which represented the characteristics of the catechol group in PDA (Fig. 2A). UV–vis spectroscopy demonstrated the successful ligation of CS to MTX and the effective encapsulation of DA. X-ray photoelectron spectroscopy analysis of the lyophilized CM@MnO_2_ NPs verified the existence of Mn, C, O, and N atoms in the NPs (Fig. S2). There were mainly 4 peaks that belonged to Mn 2p (641.07), O 1s (532.08), N 1s (399.1), and C 1s (284.3) eV. The presence of Mn 2p_1/2_ and Mn 2p_3/2_ in CM@MnO_2_ NPs was further demonstrated by high-resolution Mn spectra. In addition, the measured spin–orbit splitting distance was about 11.7 eV between the Mn 2p_1/2_ and Mn 2p_3/2_ peaks, indicating the presence of MnO_2_ in CM@MnO_2_ NPs (Fig. 2B).

The particle sizes of CM NPs, CM@MnO_2_ NPs, and PCM@MnO_2_ NPs were (269.5 ± 9.1), (151.3 ± 1.8), and (184.9 ± 2.2) nm, respectively (Fig. 2C to E and Table S1). Compared to that of CM NPs, the particle sizes of CM@MnO_2_ NPs and PCM@MnO_2_ NPs decreased after being decorated with PDA and Mn^2+^, which compacted the nanostructure through interaction forces with MTX [34,35]. Transmission electron microscopy showed that the PCM@MnO_2_ NPs possessed a uniform size and a spherical appearance (Fig. 2F). The zeta potential of PCM@MnO_2_ NPs ((−28.8 ± 1.9) mV) was elevated compared to that of CM@MnO_2_ NPs ((−16.0 ± 1.2) mV), indicating that PDA was successfully modified in NPs (Fig. 2G and Table S1).

The encapsulation efficiency (EE%) of MTX from CM NPs, CM@MnO_2_ NPs, and PCM@MnO_2_ NPs was (55.72 ± 2.76)%, (71.40 ± 1.07)%, and (73.6 ± 1.0)%, respectively (Fig. 2H and Table S1). The results indicated that the EE% of PCM@MnO_2_ NPs significantly increased. This was because MnCl_2_ was added to CS, and manganese ions could bind to CS chains via sulfate or carboxyl groups (polysaccharide–metal complexes). In addition, Mn^2+^ could further coordinate with MTX. The introduction of Mn^2+^ could compact the nanostructure and increase the stability of NPs and improve the EE% of MTX. CM NPs, CM@MnO_2_ NPs, and PCM@MnO_2_ NPs were added to PBS (containing 10% FBS) and incubated at 4 °C to observe serum stability and placement stability (Fig. 2I and Fig. S3). The particle size, zeta potential, and PDI of PCM@MnO_2_ NPs in PBS (containing 10% FBS) at 4 °C were monitored, and slight changes were found over 4 d. In addition, no aggregation was detected in the stability test, suggesting that the serum stability of NPs was satisfactory. However, CM@MnO_2_ NPs dispersed in PBS would cause agglomeration and sedimentation. The PDA shell improved the biocompatibility and decreased the drug leakage (Fig. 2J) [22].

### Cumulative drug release of MTX

The drug release curve of MTX was detected by dialysis. As shown in Fig. 2K, the release of MTX in PCM@MnO_2_ NPs was related to pH. In the arthritis weakly acid microenvironment, the pH is 6.5, and pH 5.2 is the acidic condition within M1 macrophages [36,37]. The cumulative release of MTX at pH 7.4 and 5.2 after 48 h was about (61.59 ± 0.7)% and (82.2 ± 1.3)%, respectively. To further validate the reaction of the NPs under acidic pH conditions, TA assay was performed. It was discovered that the mixture of TA and H_2_O_2_ gave rise to fluorescence at 360 nm. In the presence of TA, PCM@MnO_2_ NPs were added to different pH and H_2_O_2_ (1 mM) mixtures (Fig. S4). The fluorescence intensity of TA decreased as the acidic pH decreased, which further confirmed the pH-responsive degradation behavior of PCM@MnO_2_ NPs. The NPs degraded under acidic conditions and further increased drug release.

### Decomposition of H_2_O_2_ and production of oxygen

**Fig. 3.**
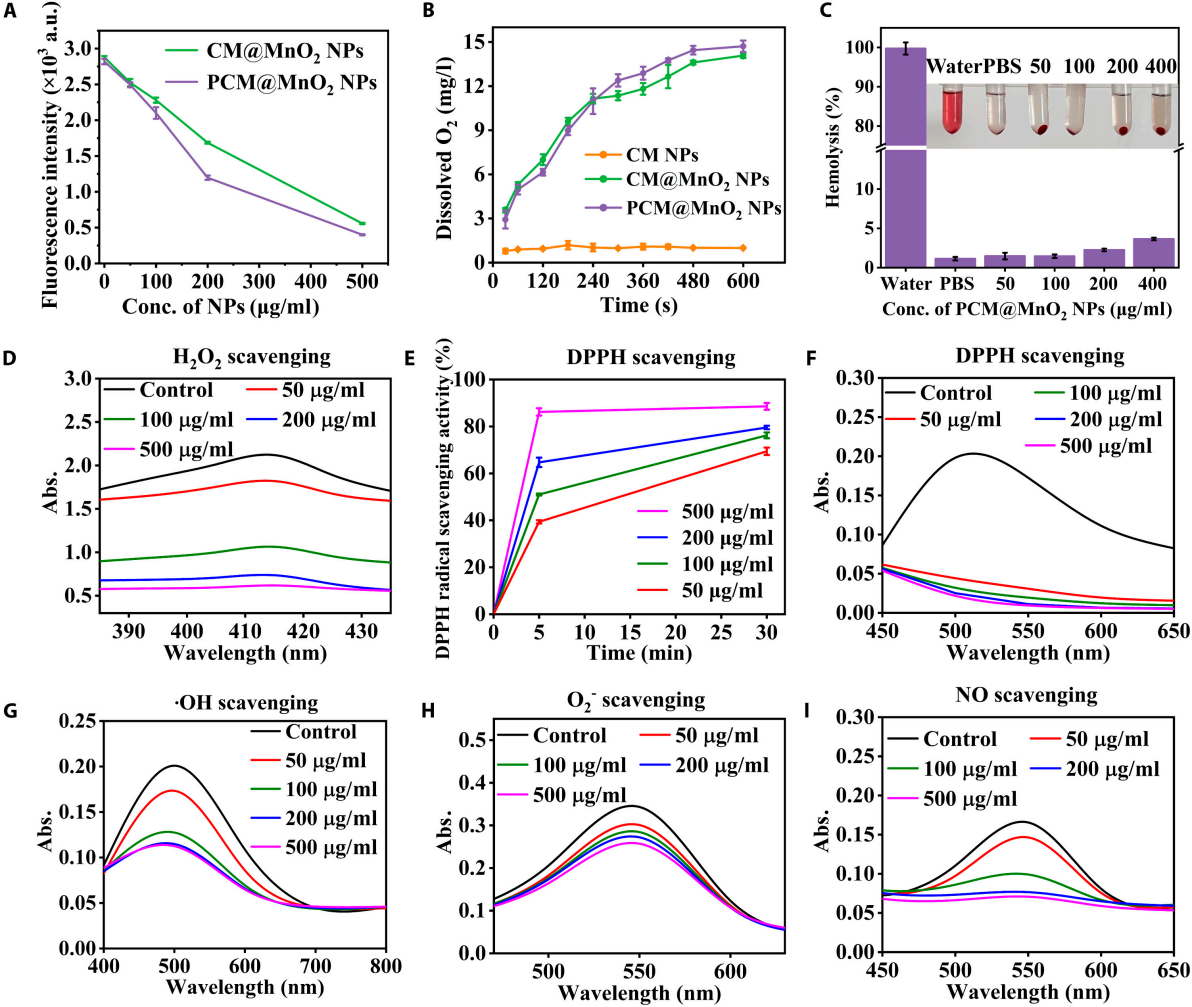
ROS scavenging capability of PCM@MnO_2_ NPs. (A) Terephthalic acid–peroxide scavenging test of CM@MnO_2_ NPs and PCM@MnO_2_ NPs with different concentrations. (B) Generation curves of O_2_ generated by MTX-loaded nanoparticles in 1 mM H_2_O_2_ solution. (C) The hemolysis percentage of PCM@MnO_2_ NPs. (D) UV–vis spectra and the kinetic curves showing the H_2_O_2_ scavenging activity of PCM@MnO_2_ NPs. (E) UV–vis spectra and (F) the kinetic curves showing the activity of PCM@MnO_2_ NPs with different concentrations to 2,2-diphenyl-2-picrylhydrazyl (DPPH) scavenging. UV–vis spectra of PCM@MnO_2_ NPs with different concentrations for scavenging (G) ·OH, (H) O_2_^−^, and (I) NO.

To evaluate the peroxide scavenging capacity of different NPs, CM@MnO_2_ NPs and PCM@MnO_2_ NPs were added to a mixture of TA and H_2_O_2_. After mixing TA with H_2_O_2_, the fluorescence of TA was detected at 360 nm, and the fluorescence intensity decreased significantly with the increase in the preparation concentration (Fig. 3A). Moreover, PCM@MnO_2_ NPs had a lower fluorescence intensity than CM@MnO_2_ NPs at the same concentration. The results indicated that PDA could play an enhanced role in the elimination of H_2_O_2_ (Fig. 3A). MnO_2_ possesses a peroxidase-like activity and can break down H_2_O_2_ to produce water and oxygen. Several studies have indicated that PDA can be used not only as a reducing agent for redox reactions but also as a catalyst for H_2_O_2_ decomposition [38]. Dissolved oxygen was measured to further evaluate the peroxidase activity of CM@MnO_2_ NPs and PCM@MnO_2_ NPs. CM@MnO_2_ NPs and PCM@MnO_2_ NPs could rapidly generate oxygen in a short time, and the measured dissolved oxygen values after 300 s were 11.4 and 12.8 mg/l, respectively (Fig. 3B). The oxygen content of CM NPs at the same concentration did not increase significantly. These results showed that CM@MnO_2_ NPs and PCM@MnO_2_ NPs possessed peroxidase-like activities and could efficiently decompose H_2_O_2_ to produce oxygen. In hemolysis studies, no erythrocyte lysis was found after treatment with CM@MnO_2_ and PCM@MnO_2_ NPs in mouse blood (Fig. 3C and Fig. S5). The carrier material had good biocompatibility.

### ROS scavenging capability of PCM@MnO_2_ NPs

PCM@MnO_2_ NPs with different concentrations (50, 100, 200, and 500 μg/ml) were mixed with DPPH ethanol (0.1 mM) to assess the ROS scavenging ability. The solution color changed from purple to light yellow (Fig. 3E and F and Fig. S6) with the increase in PCM@MnO_2_ NP concentration. DPPH clearance increased with the increase in the concentration of PCM@MnO_2_ NPs. After 30 min, the absorbance of PCM@MnO_2_ NPs (500 μg/ml) became the lowest at 450 to 650 nm, which indicated that PCM@MnO_2_ NPs could effectively scavenge ROS due to the superior antioxidant activity of PDA and the consumption of H_2_O_2_ by the MnO_2_ reaction. After incubation with various concentrations of PCM@MnO_2_ NPs, the absorption peaks of the yellow complexes of H_2_O_2_ and titanium sulfate gradually decreased at 415 nm with increasing concentrations (Fig. 3D), indicating that PCM@MnO_2_ NPs effectively reduced H_2_O_2_ levels.

Based on the above experimental results, the related radicals including O_2_^−^, ·OH, and NO were further investigated. The corresponding kits were added for O_2_^−^, ·OH, and NO_2_^¯^ determinations to generate UV–vis absorbance signals. The results showed that due to the dual effect of PDA and MnO_2_, the NPs demonstrated concentration-dependent scavenging of multiple ROS (Fig. 3G to I). Overall, PCM@MnO_2_ NPs could scavenge a broad spectrum of ROS.

### Cytotoxicity and in vitro biocompatibility studies

**Fig. 4.**
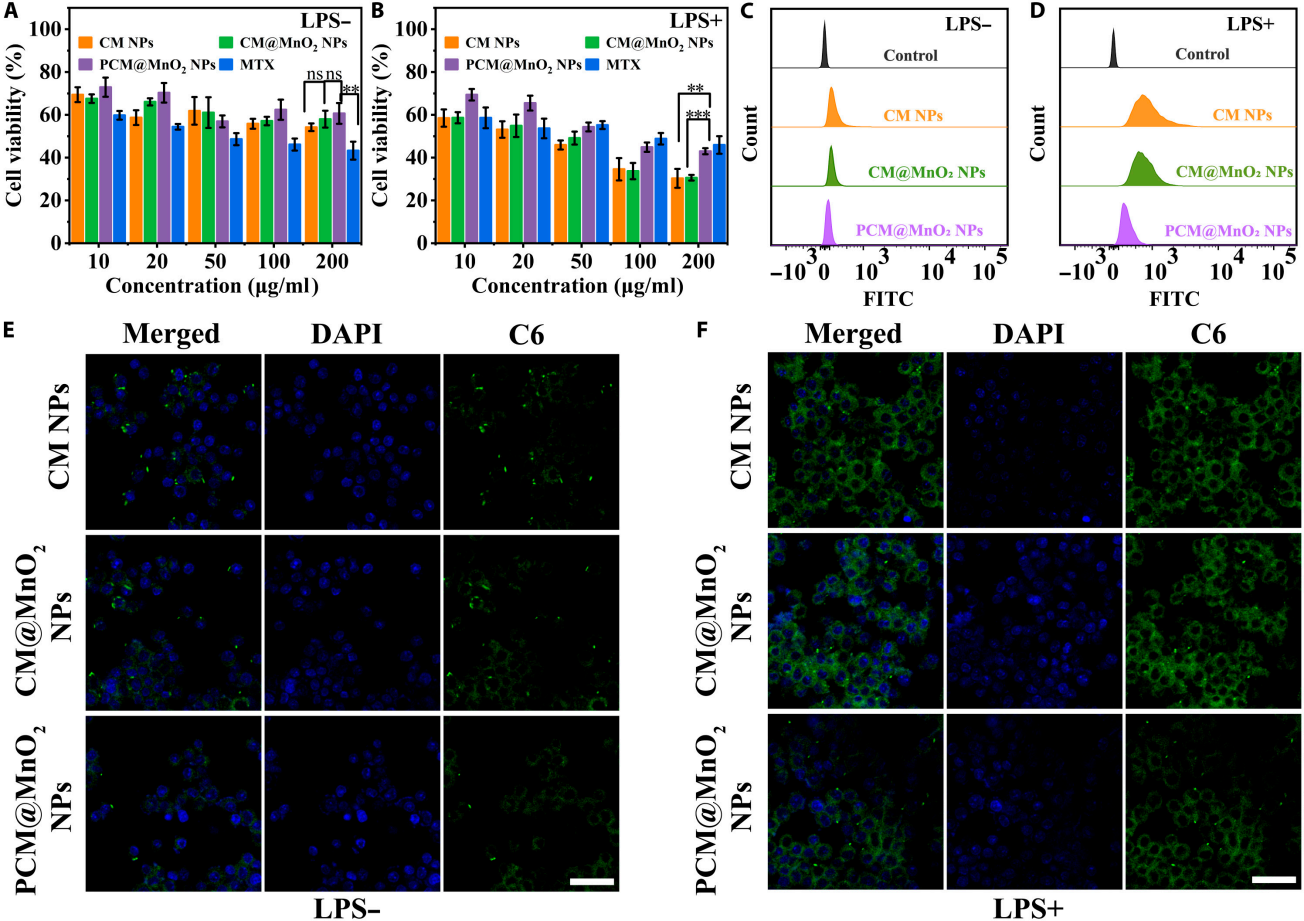
Cytotoxicity and cellular uptake of PCM@MnO_2_ NPs. The cell viability of (A) normal and (B) induced RAW264.7 cells’ incubation with MTX, CM NPs, CM@MnO_2_ NPs, and PCM@MnO_2_ NPs for 24 h (*n* = 3). Data were analyzed by one-way ANOVA (**P < 0.01; ***P < 0.001; ns expresses no significant difference). (C) Flow cytometry (FCM) to analyze the uptake ability of normal and (D) lipopolysaccharide (LPS)-induced RAW264.7 cells after treatment with CM NPs, CM@MnO_2_ NPs, and PCM@MnO_2_ NPs encapsulated C6. Scale bar = 100 μm. (E) Confocal laser scanning microscopy (CLSM) images to research the uptake ability of normal and (F) LPS-induced RAW264.7 cells after treatment with CM NPs, CM@MnO_2_ NPs, and PCM@MnO_2_ NPs encapsulated C6 (green). The nuclei were stained with 4′,6-diamidino-2-phenylindole (DAPI; blue). Scale bar = 100 μm. FITC, fluorescein isothiocyanate.

The toxic effects of CM NPs, CM@MnO_2_ NPs, and PCM@MnO_2_ NPs on pristine and activated RAW264.7 cells were analyzed. Figure 4A and B show that pristine and activated RAW264.7 cells were treated with NPs exhibited concentration-dependent cytotoxicity (Fig. S7 shows normal and successfully induced RAW264.7 cells’ morphology under the microscope). Free MTX exhibited significant cytotoxicity while inhibiting the functions of macrophages. However, formulating MTX into PCM@MnO_2_ NPs could remarkably enhance the biocompatibility (Fig. 4A). Notably, induced RAW264.7 cells were more sensitive to NPs due to targeting nanosystems. NPs delivered MTX through CS–CD44, which exhibited strong cytostatic activity by inhibiting dihydrofolate reductase activity [39,40]. Enhanced efficacy in inhibiting M1 macrophage activity was exhibited by CM NPs and CM@MnO_2_ NPs due to the high affinity for CD44 receptors. PCM@MnO_2_ NPs were less inhibitive than CM NPs and CM@MnO_2_ NPs (Fig. 4B). This was because the PDA was coated; it first dissociated in the inflammatory macrophages’ environment and then was internalized by cells for sustained drug release.

### Cellular uptake targeting

FCM and CLSM were used to study the uptake capability of pristine and LPS-induced RAW264.7 cells for various NPs. FCM and quantitative results suggested that after LPS induction, the fluorescence intensity of C6 was significantly stronger than that in normal cells (Fig. 4C and D and Fig. S8). Figure 4E and F show that the fluorescence intensity of NPs in M1 macrophages was higher than that in corresponding pristine macrophages, indicating that the uptake of NPs by activated macrophages was significantly increased. This was attributed to the high affinity between CD44 overexpressed on the surface of inflammatory macrophages and CS. Moreover, the fluorescence intensity of PCM@MnO_2_ NPs was weaker than that of CM NPs and CM@MnO_2_ NPs (Fig. 4D and F). This was due to the modification of PDA on the surface, and it affected the uptake of PCM@MnO_2_ NPs by M1 macrophages, whereas in the low-pH and high-ROS environments of the RA joints, the NPs dissociated and the exposed CS further bound to CD44 into M1 macrophages [10,11].

### Antioxidant activity of PCM@MnO_2_ NPs

**Fig. 5.**
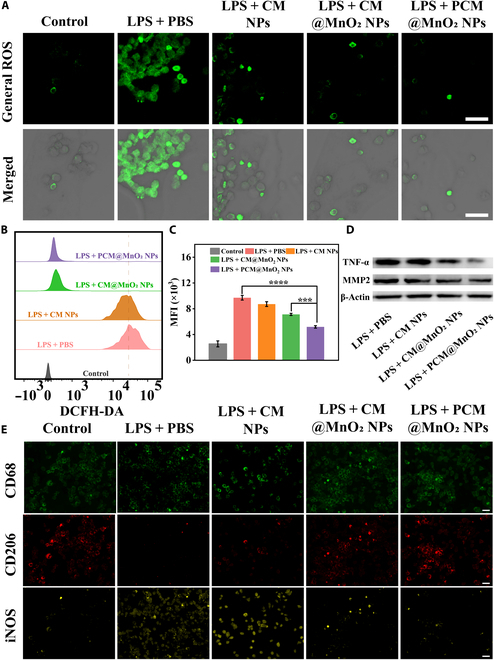
ROS scavenging and macrophages’ repolarization activity of PCM@MnO_2_ NPs. (A) The intracellular ROS levels in RAW264.7 cells were observed under CLSM; scale bar = 100 μm. (B) FCM results showing the general ROS scavenging activity of CM NPs, CM@MnO_2_ NPs, and PCM@MnO_2_ NPs. (C) The quantitative results of fluorescence intensity of 2′,7′-dichlorodihydrofluorescein diacetate (DCFH-DA). (D) Protein expression of TNF-α and matrix metalloproteinase-2 (MMP2) in LPS-induced RAW264.7 cells after different treatments. (E) Immunofluorescence staining images of CD68, inducible nitric oxide synthase (iNOS), and CD206 of LPS-activated RAW264.7 cells treated with different nanoparticles. Scale bar = 100 μm. Data expressed as mean ± SD (*n* = 3) (***P < 0.001, and ****P < 0.0001) and analyzed by one-way ANOVA. MFI, mean fluorescence intensity.

Studies have shown that the ROS level in the inflammatory microenvironment was significantly increased and was strongly related to the mechanism of RA. The overproduced ROS could not only induce an imbalance in chondrocytes metabolism but also increase the level of pro-inflammatory cytokines and further activate macrophages into the M1 phenotype. Therefore, it was essential to detect the ROS clearance capacity of NPs for treating RA. The antioxidant activity of NPs on cells was investigated by using fluorescent probes. The DCFH-DA radical probe was added, and M1 macrophages showed high ROS levels. After treatment with NPs, the fluorescence was weakened to varying degrees, indicating that the NPs had a universal ROS elimination activity (Fig. 5A). FCM and quantitative results are shown in Fig. 5B and C; the PCM@MnO_2_ NP treatment group had the lowest ROS fluorescence intensity. This was because PDA had excellent antioxidant activity and MnO_2_ could decompose H_2_O_2_.

Under the dual action of PDA and MnO_2_, PCM@MnO_2_ NPs possessed the potent ability to scavenge ROS and reduce cellular oxidative damage, which could effectively alleviate the progression of RA.

### Anti-inflammatory and chondroprotective effects of PCM@MnO_2_ NPs

An ELISA kit was used to detect the levels of TNF-α and IL-1β in different groups. The results indicated that NPs could efficiently inhibit these cytokines and PCM@MnO_2_ NPs possessed the most effective inhibition (Fig. S9). These results showed that PCM@MnO_2_ NPs could efficiently scavenge ROS, reduce the secretion of inflammatory factors, and ultimately alleviate the development of RA. To further confirm the anti-inflammatory ability of NPs, TNF-α and MMP2 in M1 macrophages were explored using western blot. The results indicated that NPs could effectively inhibit TNF-α, and PCM@MnO_2_ NPs possessed the most effective inhibition. Notably, excessive ROS and inflammatory cytokines can trigger the secretion of matrix metalloproteinases (MMPs), which can lead to cartilage and bone damage at the RA [41–43]. The level of MMP2 was higher for activated RAW264.7 cells. However, MMP2 was significantly down-regulated after being treated by PCM@MnO_2_ NPs due to its cytokine inhibitory activity (Fig. 5D). Quantitative western blot analysis results were consistent with the above data (Fig. S10). PCM@MnO_2_ NPs exhibited the potential to protect cartilage and bone from MMPs by efficiently clearing ROS and inhibiting the expression of RA inflammatory factors. Regarding regeneration, CS primarily facilitates cartilage regeneration by stimulating chondrocyte proliferation and differentiation [44]. Compared with CS, PCM@MnO_2_ NPs exerted a negligible effect on chondrocyte proliferation (Fig. S11). It might be because MTX curbs cell growth. Therefore, PCM@MnO_2_ NPs predominantly facilitated cartilage reparation via a remodeling microenvironment. It was due to PCM@MnO_2_ NPs efficiently eliminating ROS, restraining inflammatory factor secretion, and reducing MMPs expression.

### Repolarization activity of PCM@MnO_2_ NPs

In RA, macrophages are often recruited and predominantly differentiated into the M1 phenotype, which in turn promotes the progression of RA. It has been reported that hypoxia and excess ROS play a crucial role in the process of macrophage polarization. They can form an inflammatory cascade that will amplify inflammation and eventually lead to a vicious cycle [45]. Therefore, the effect of PCM@MnO_2_ NPs on macrophage reprogramming was evaluated by ameliorating hypoxia and reducing ROS in vitro. Macrophage phenotypes were characterized by immunofluorescence staining of CD68, iNOS (representing M1 marker, yellow), and CD206 (representing M2 marker, red). After LPS stimulation, RAW264.7 cells exhibited bright iNOS fluorescence, whereas CD206 fluorescence was weaker, confirming successful M1 polarization. However, after incubation with PCM@MnO_2_ NPs, the yellow fluorescence intensity of iNOS was weakened and the red fluorescence intensity of CD206 was enhanced, indicating that the macrophage phenotype undergoes M1–M2 repolarization (Fig. 5E). MnO_2_ catalytically converts H_2_O_2_ to O_2_ while PDA scavenges ROS, synergistically alleviating hypoxia and oxidative stress in RA. In summary, PCM@MnO_2_ NPs improved the RA microenvironment through multiple actions, thereby further promoting the repolarization of macrophages from M1 to M2.

### Targeted delivery of individual NPs in CIA mice

**Fig. 6.**
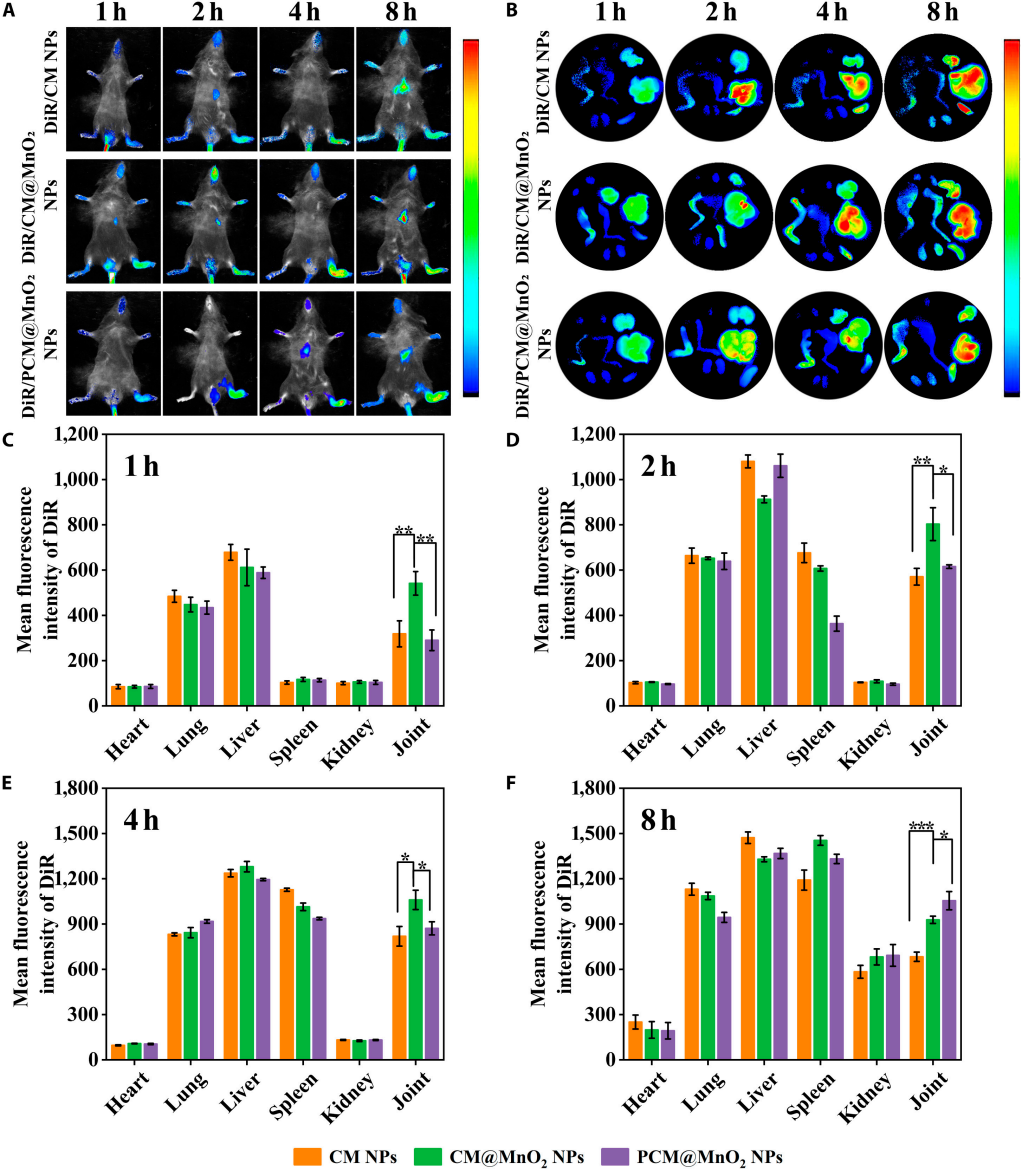
Targeting capacity of PCM@MnO_2_ NPs. (A) In vivo biodistribution of different preparations in CIA mice. (B) In vitro photos of dissected tissues from CIA mice: (clockwise) heart, lung, liver, spleen, kidney, and joint. (C to F) The quantitative of biological analysis of representative organs and joints at 1, 2, 4, and 8 h. Data expressed as mean ± SD (*n* = 3) (*P < 0.05, **P < 0.01, and ***P < 0.001) and analyzed by one-way ANOVA.

The in vivo targeting ability of PCM@MnO_2_ NPs was investigated using a mouse model of CIA. DiR-labeled CM NPs, CM@MnO_2_ NPs, and PCM@MnO_2_ NPs were intravenously injected, and fluorescence was monitored with an in vivo imaging system (1, 2, 4, and 8 h) after administration. It could be observed from Fig. 6A that inflammatory joints appeared and retained strong fluorescence signal, and then the fluorescence intensity of the joints was quantified. After intravenous injection, the accumulation of targeted CM NPs, CM@MnO_2_ NPs, and PCM@MnO_2_ NPs in the site of inflammation gradually increased with time and the joint fluorescence intensity of CM NPs and CM@MnO_2_ NPs reached the strongest at 4 h. CM@MnO_2_ NPs accumulated more in joints than CM NPs at 4 h because in addition to targeting macrophages through CD44, NPs also passively targeted RA sites with more appropriate particle sizes [46]. However, PCM@MnO_2_ NPs had a certain role in prolonging the in vivo circulation of NPs due to strong electrostatic repulsion and excellent bioadhesion of PDA, so the fluorescence intensity of PCM@MnO_2_ NPs at 8 h was significantly stronger than those of the CM NP and CM@MnO_2_ NP groups. After 8 h of administration, the main organs were obtained for in vitro observation (Fig. 6B) and the fluorescence intensity was further quantified (Fig. 6C to F) [47]. Accumulation of DiR-labeled NPs was apparently observed in the liver and spleen, which was because most intravenous NPs were trapped in the mononuclear phagocyte system, such as the liver and spleen, during circulation in the body. In addition, we also observed a high fluorescence intensity in the lungs, which might be due to arthritis and inflammation of the lungs that led to lung involvement.

### In vivo therapeutic effect of NPs on CIA mice

#### Evaluation of joint indicators in CIA mice

**Fig. 7.**
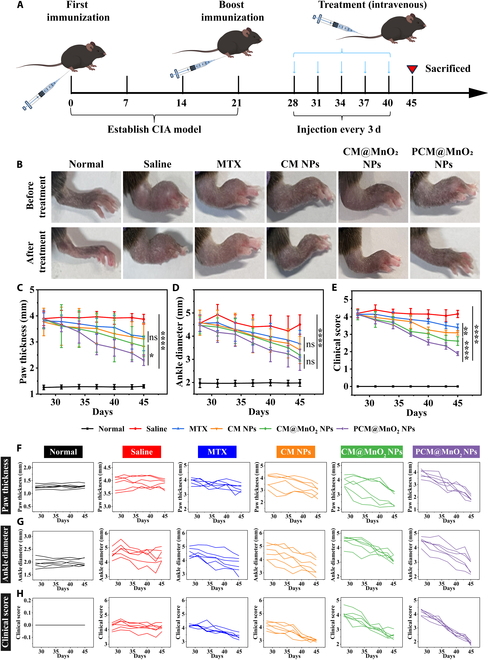
Pharmacodynamic study of various preparations in CIA mice. (A) Schematic diagram of induction of the CIA mouse model and intravenous treatment of each preparation. (B) Corresponding photographs of the inflamed joints of CIA mice administered with saline, MTX, CM NPs, CM@MnO_2_ NPs, and PCM@MnO_2_ NPs. (C) Measurements of hind paw thickness in CIA mice, (D) changes in ankle diameter after different treatments, and (E) evaluation of arthritis joint scores in CIA mice. (F) Changes in hind paw thickness measured during treatment for each mouse. (G) Ankle diameter of each mouse during treatment. (H) Trends in the arthritis scores of each mouse during treatment. The above experimental results are expressed as mean ± SD (*n* = 6) (**P < 0.01, ***P < 0.001, and ****P < 0.0001; ns expresses no significant difference) and were analyzed by one-way ANOVA.

CIA mice were injected with different preparations, including saline, CM NPs, CM@MnO_2_ NPs, and PCM@MnO_2_ NPs, every 3 d starting on day 28 to evaluate the antirheumatic properties of NPs (Fig. 7A). Paw swelling, ankle diameter, and clinical joint scores were used to determine arthritis severity in RA mice. It could be intuitively seen from the photos that the swelling degree of the paw and the diameter of the ankle joint of the mice injected with each preparation were reduced compared with those of the saline group (Fig. 7B). The severity of PCM@MnO_2_ NPs was most significantly alleviated (Fig. 7B). Compared with those of other administration groups, the clinical score of CIA mice in the saline group was the highest and showed a certain upward trend during treatment (Fig. 7C to E). Among all treatment groups, CIA mice administered with PCM@MnO_2_ NPs showed the most remarkable reduction in joint score and scores closest to those of normal mice (Fig. 7E). During treatment, paw swelling and ankle diameter were reduced after administration of CM NPs, CM@MnO_2_ NPs, and PCM@MnO_2_ NPs (Fig. 7C and D). The increasing trend of hind paw thickness and ankle diameter during treatment was similar in all mice (Fig. 7F and G). Compared with the MTX group, NPs showed stronger inhibitory effects on different indexes of RA joints. Moreover, the experimental results showed that PCM@MnO_2_ NPs had the most significant therapeutic effect on CIA mice (Fig. 7F to H). NPs could better accumulate passively into the inflammatory synovial tissue through the extravasation through leaky vasculature and subsequent inflammatory cell-mediated sequestration (“ELVIS”) effect and trigger the disintegration of NPs in the RA microenvironment [48]. After PDA degradation, CS was exposed and targeted CD44 on macrophages, which further improved the anti-inflammatory ability. In addition, to clearly demonstrate the efficacy of PCM@MnO_2_ NPs as delivery biomaterials, the therapeutic effect of CS without NPs in CIA mice was investigated. After CS treatment, no significant relief of joint swelling was observed and the joint scores of individual mice failed to decrease markedly. CS alone demonstrated relatively weak efficacy, and the effect in reducing joint scores was significantly inferior to that of MTX (Fig. S12). This was because the limited mechanism of action and slow onset of CS. Therefore, CS is often used as a carrier to deliver some antirheumatic drugs for more effective treatment of RA.

#### Histological analysis

**Fig. 8.**
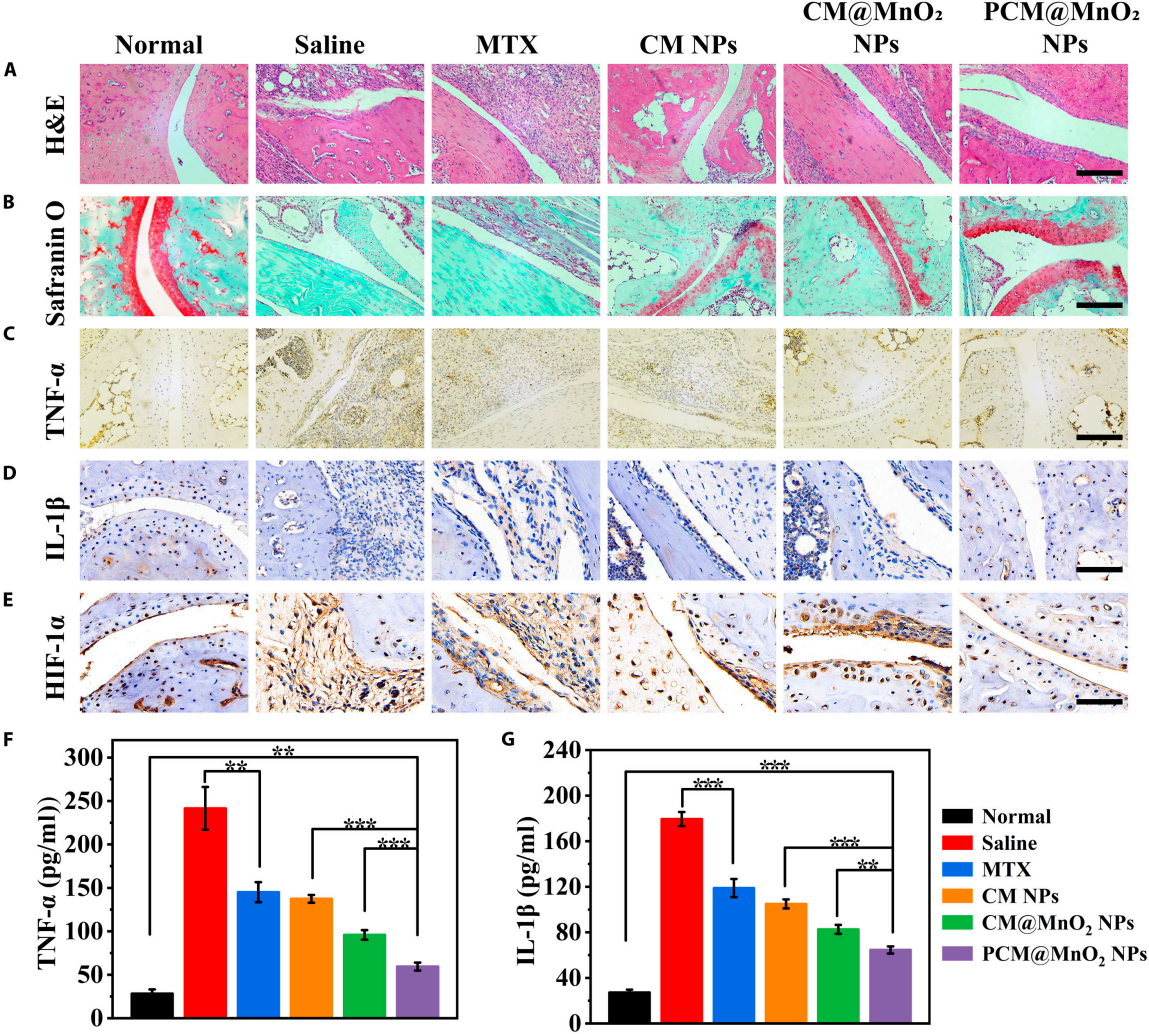
The histopathology of the inflamed joint was evaluated by histological analysis. (A) hematoxylin–eosin (H&E), (B) Safranin O–fast green staining, (C) TNF-α, (D) IL-1β, and (E) HIF-1α immunohistochemistry; scale bars = 200 μm. The serum levels of (F) TNF-α and (G) IL-1β were detected in each group by an enzyme-linked immunosorbent assay (ELISA) kit (*n* = 3) (**P < 0.01 and ***P < 0.001) and analyzed by one-way ANOVA.

To further demonstrate the efficacy of NPs, histological analysis and immunohistochemistry were performed on arthritic joints. As shown in the H&E section results in Fig. 8A, severe synovial hyperplasia and cartilage damage were found in the saline group. Compared with the saline group, free MTX could alleviate cartilage injury to a certain extent, but CM@MnO_2_ NPs and PCM@MnO_2_ NPs displayed better treatment effects on articular cartilage injury. This was because CM@MnO_2_ NPs and PCM@MnO_2_ NPs could be more targeted and aggregated in cartilage through the RA microenvironment, which could better exert anti-inflammatory and cartilage-protective effects. The SO-FG staining results of the saline group (Fig. 8B) showed that the joint staining area was the smallest, indicating that the cartilage destruction was serious, and the damage was alleviated after free MTX treatment. It was obvious that the joint Safranin O staining area in the PCM@MnO_2_ NP treatment group was larger. The effect was better than those of CM NPs and CM@MnO_2_ NPs. PCM@MnO_2_ NPs exhibited enhanced ROS scavenging capacity, attributable to the synergistic effect of PDA and MnO_2_. The results of SO-FG and H&E staining corresponded to each other, which further verified that CM@MnO_2_ NPs and PCM@MnO_2_ NPs had excellent antioxidant activity and synergistically improved the RA microenvironment more efficiently.

The effect of NPs on macrophage repolarization in inflamed joints was determined. Compared with the saline group with prominent M1 features, CM NPs, CM@MnO_2_ NPs, and PCM@MnO_2_ NPs more effectively suppressed inflammatory macrophages via targeted delivery of MTX to the RA joint, as evidenced by the robust immunofluorescence signal of iNOS (the M1 marker, yellow). Nevertheless, in contrast to CM@MnO_2_ NPs and PCM@MnO_2_ NPs, CM NPs exhibited a less pronounced effect on M2 repolarization. After PCM@MnO_2_ NP treatment, the high fluorescence intensity of CD206 (the M2 marker, red) was demonstrated (Fig. S13), attributable to the efficient scavenging of ROS at inflamed joints by PDA and MnO_2_, leading to O_2_ generation and mitigation of the RA microenvironment.

#### Cytokine determination

It has been reported that there is a positive feedback relationship between pro-inflammatory cytokines and cartilage destruction and bone erosion. Therefore, it is also necessary to monitor the levels of TNF-α and IL-1β in histological sections. Figure 8C and D show that MTX, CM@MnO_2_ NPs, and PCM@MnO_2_ NPs all effectively reduced the expression of TNF-α and IL-1β compared with the saline group. HIF-1α, which is strongly associated with the inflammatory response, was also tested. The expression of HIF-1α was all decreased after treatment with all preparations, while the decrease was most significant in the PCM@MnO_2_ NP group (Fig. 8E). This phenomenon was due to the synergistic catalytic effect of PDA and MnO_2_ on the production of O_2_ from H_2_O_2_. In addition, an ELISA kit was used to further measure the levels of TNF-α and IL-1β in serum. The lowest levels of inflammatory cytokines were found in the serum of CIA mice administered with PCM@MnO_2_ NPs, which further validated the strong anti-inflammatory effect of PCM@MnO_2_ NPs (Fig. 8F and G), mainly because PCM@MnO_2_ NPs could alleviate the inflammatory cascade caused by oxidative stress and hypoxia by scavenging ROS and producing O_2_. These results all suggested that PCM@MnO_2_ NPs with synergistic anti-inflammatory effects could effectively inhibit the progression of RA disease.

### In vivo safety of PCM@MnO_2_ NPs

**Fig. 9.**
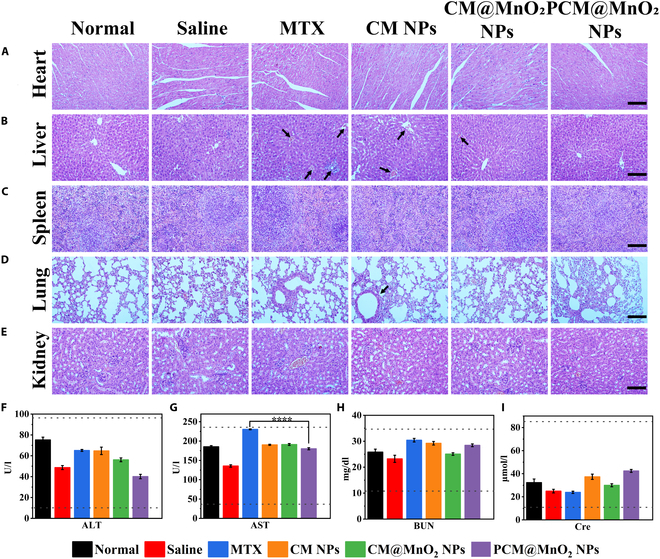
Safety evaluation of PCM@MnO_2_ NPs. Histopathological analysis of major organs: heart, liver, spleen, lung, and kidney. H&E staining on the (A) heart, (B) liver (black arrows represent congestion and dilation of the hepatic sinusoids), (C) spleen, (D) lung (black arrow indicates enlargement of the alveolar space), and (E) kidney from each group of CIA mice that were treated with normal, saline, MTX, CM NP, CM@MnO_2_ NP, and PCM@MnO_2_ NP treatments. Scale bars = 200 μm. (F and G) Hepatotoxicity of each formulation by the determination of alanine aminotransferase (ALT) and aspartate aminotransferase (AST) levels. (H and I) Nephrotoxicity of various preparations by examining blood urea nitrogen (BUN) and creatinine (Cre) levels. Results are expressed as mean ± SD (*n* = 3) (****P < 0.0001) and analyzed by one-way ANOVA.

The safety of PCM@MnO_2_ NPs in CIA mice was evaluated, as MTX has been reported to induce strong side effects at high doses over a long period of time. No significant change in weight was observed during treatment (Fig. S14), indicating that none of the preparations had short-term toxicity in CIA mice. Moreover, to further evaluate the toxicity to liver and kidney, the levels of ALT, AST, BUN, and Cre were detected (Fig. 9F to I). AST was significantly elevated after treatment with MTX, suggesting possible hepatotoxicity, whereas those of groups treated with CM NPs, CM@MnO_2_ NPs, and PCM@MnO_2_ NP were in the normal range (Fig. 9G), indicating that PCM@MnO_2_ NPs had no side effects on the liver. BUN and Cre were used to study nephrotoxicity, and no significant changes were found. Histological analyses of major organs were performed to further evaluate the safety of CM NPs, CM@MnO_2_ NPs, and PCM@MnO_2_ NPs. H&E results are shown in Fig. 9A to E; no heart tissue damage was observed after treatment with different preparations (Fig. 9A). Figure 9B shows that after administration with MTX, the central vein and surrounding hepatic venous sinuses of CIA mice were dilated and congested, and the hepatocyte nucleus became smaller and darker, indicating that the hepatocytes were damaged [49]. However, there was a significant improvement in liver damage after CM@MnO_2_ NP and PCM@MnO_2_ NP administration. This was because CM@MnO_2_ NPs and PCM@MnO_2_ NPs could deliver MTX to the lesion site more precisely. RA was usually accompanied by inflammatory cell infiltration in the lungs, the alveolar cavity was enlarged or fused to form alveoli. The lung damage was not changed in the MTX group, whereas it was significantly improved in the CM@MnO_2_ NPs and PCM@MnO_2_ NP groups (Fig. 9D). The results strongly suggest that PCM@MnO_2_ NPs had good biological safety and effectively improved the therapeutic index of MTX and thus was an effective nanodelivery system for targeted RA treatment.

## Conclusion

In summary, pH-responsive multifunctional PCM@MnO_2_ NPs possessed good biocompatibility, scavenging excess ROS and inhibiting the generation of pro-inflammatory cytokines. Moreover, they could also significantly improve the hypoxic microenvironment by producing oxygen and reprogram M1/M2 macrophages, thereby achieving efficient treatment of RA. PCM@MnO_2_ NPs targeted the delivery of MTX by binding to CD44 receptors, achieved intracellular precise drug release, and improved the therapeutic effect on RA. In vivo studies showed that PCM@MnO_2_ NPs could alleviate claw swelling in CIA mice, reduce inflammatory cell infiltration in lesions, and protect cartilage. In summary, compared with free MTX drugs, PCM@MnO_2_ NPs accumulated effectively in inflamed joints and had better anti-inflammatory activity and biosafety, thus providing a promising approach for the treatment of RA.

## Data Availability

The data that support the findings of this study are available in the Supplementary Materials of this article.
