# Supplementary material for: Manganese Dioxide-Based pH-Responsive Multifunctional Nanoparticles Deliver Methotrexate for Targeted Rheumatoid Arthritis Treatment
Source: Biomater Res. 2025 May 14;29:0187. doi: 10.34133/bmr.0187 (PMC12076153; doi:10.34133/bmr.0187)
Supplement: Supplementary 1 — Table S1 Figs. S1 to S14 [file bmr.0187.f1.docx]

**Supporting Information**

**Manganese dioxide-based pH-responsive multifunctional nanoparticles eliminate ROS and deliver methotrexate for targeted rheumatoid arthritis treatment**

Jingwen Jia^a,1^, Min Liu^a,1^, HanYang^a^, XiaoFang Li^a^, Siyi Liu^a^, Kexin Li^a^, Jiulong Zhang^a^*^,2^, Xiuli Zhao^a^*^,1^

^a^College of Pharmacy, Shenyang Pharmaceutical University, 103 Wenhua Road, Shenyang, Liaoning 110016, China

*Corresponding author at: College of Pharmacy, Shenyang Pharmaceutical University, 103 Wenhua Road, Shenyang, Liaoning 110016, China

*E–mail* address: raura3687yd@163.com (X. Zhao), zjl1160@163.com (J. Zhang).

^1^Co-first author.

^2^Co- corresponding author.

^1^ Jingwen Jia and Min Liu contributed equally to this work and should be regarded as co-first authors.

1. Characterization of PCM@MnO_2_ NPs

**Table S1** Particle size, polydispersity index (PDI), *Zeta* potential, encapsulation efficiency (*EE*%) and drug loading efficiency (DLE) of different NPs.

| **Formulations** | **Composition** **(*w*/*w*)** | **Size (nm)** | **PDI** | **Zeta (mV)** | **EE (%)** | **DLE (%)** |
| --- | --- | --- | --- | --- | --- | --- |
| **CM NPs** | **CS/MTX (10:5)** | **269.5 ± 9.1** | **0.189 ± 0.036** | **-22.9 ± 1.8** | **55.72 ± 2.76** | **18.57 ± 0.92** |
| **CM@MnO_2_ NPs** | **CS/MTX/MnCl_2_^.^4H_2_O**  **(10:5:5)** | **151.3 ± 1.8** | **0.225 ± 0.036** | **-16.0 ± 1.2** | **71.40 ± 1.07** | **17.85 ± 0.27** |
| **PCM@MnO_2_ NPs** | **PDA/CS/MTX/MnCl_2_^.^4H_2_O**  **(4:10:5:5)** | **184.9 ± 2.2** | **0.105 ± 0.034** | **-28.8 ± 1.9** | **73.61 ± 1.08** | **15.34 ± 0.22** |

Each value represented as mean ± SD (*n* = 3).

2. FTIR spectra


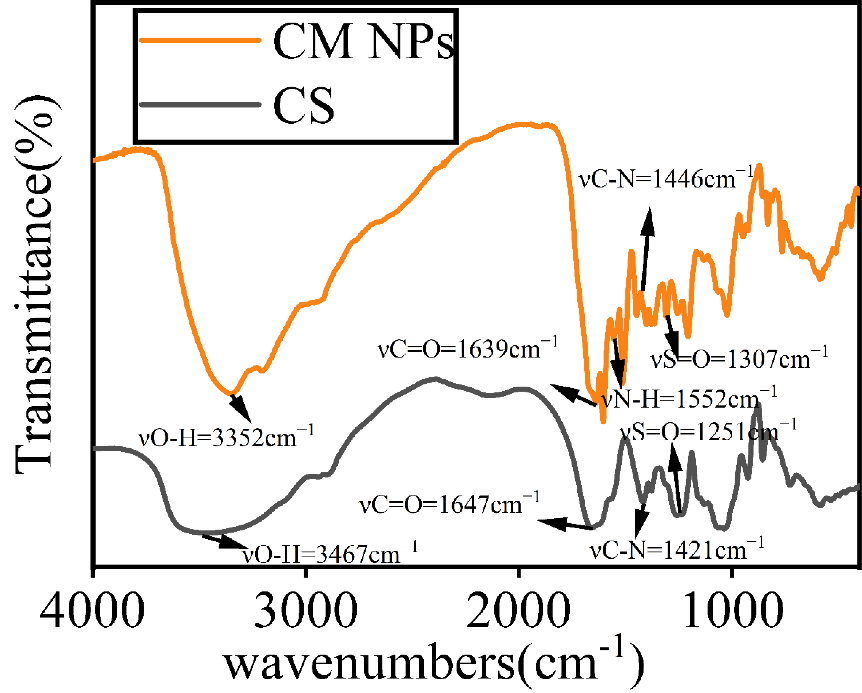


**Figure S1** FTIR spectra of CS and CM NPs.

3. XPS spectrum


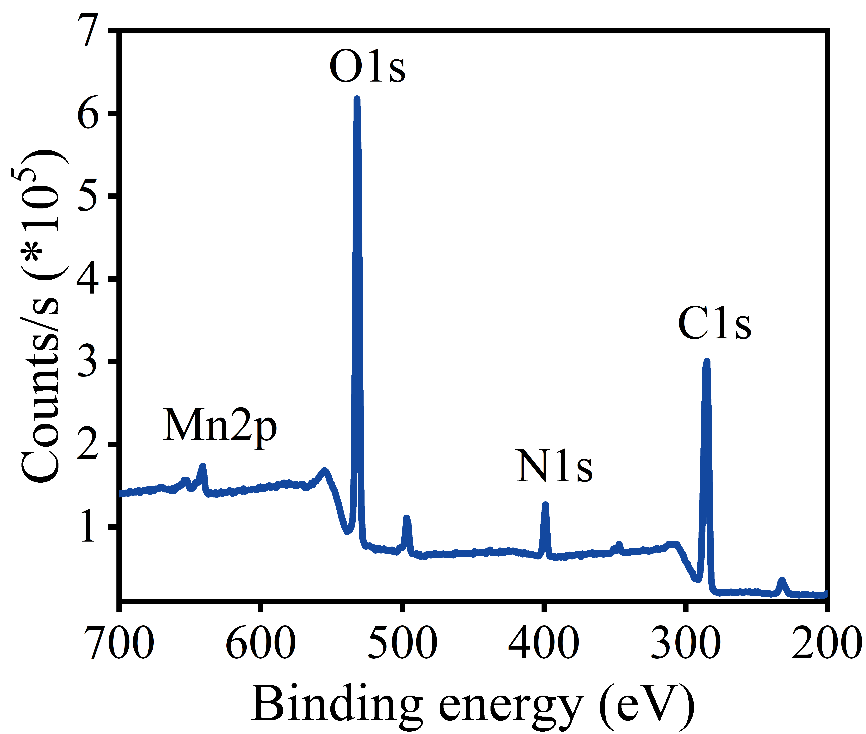


**Figure S2** XPS spectrum analysis of CM@MnO_2_ NPs.

4. Stability of NPs


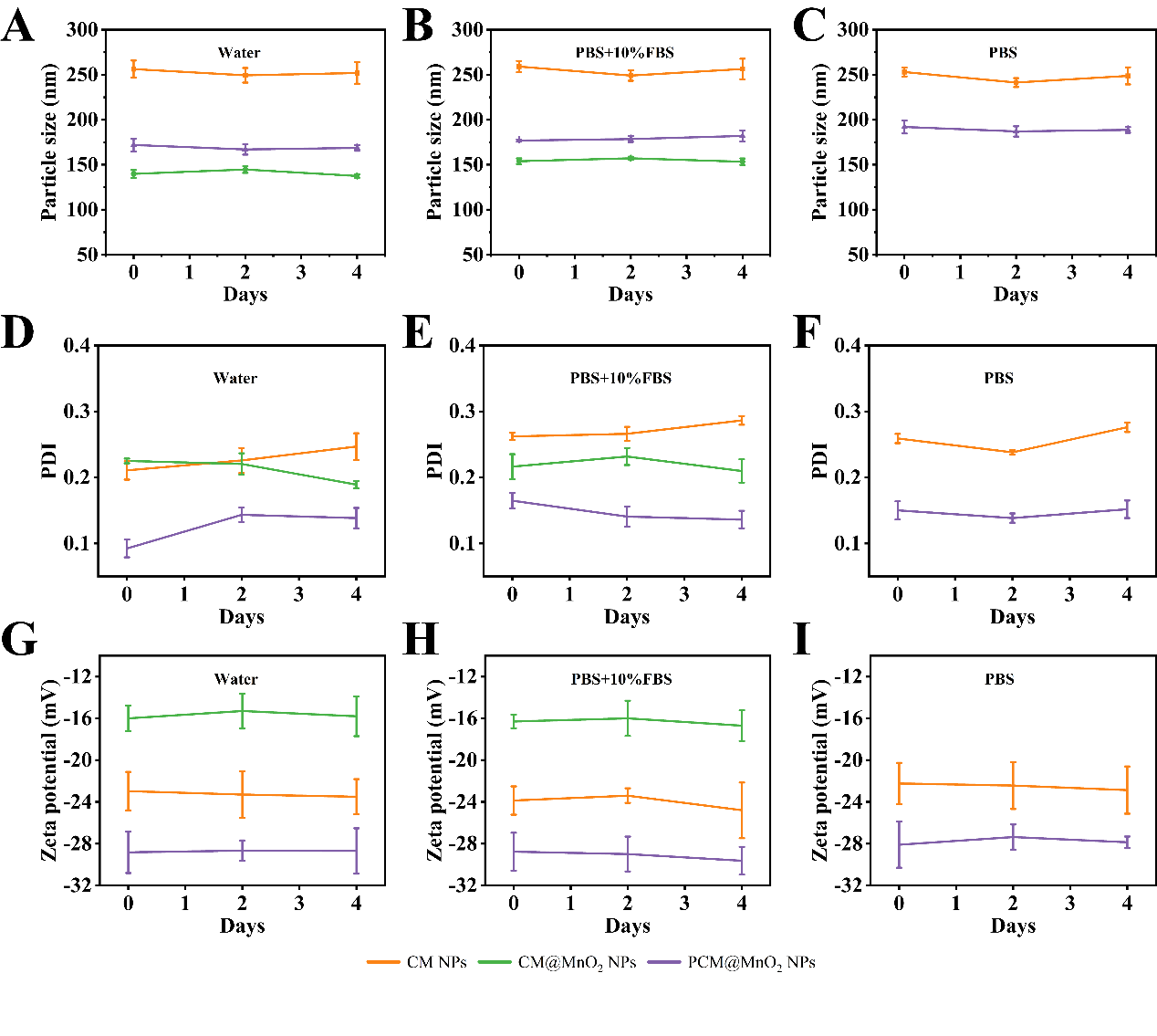


**Figure. S3**. (A-C) The Particles sizes of CM, CM@MnO_2_, and PCM@MnO_2_NPs during 4 days incubation with Water, PBS solution plus 10% FBS, and PBS. (D-F) The PDI of CM, CM@MnO_2_, and PCM@MnO_2_ NPs during 4 days incubation with Water, PBS solution plus 10% FBS, and PBS. (G-I) The Zeta potential of CM, CM@MnO_2_, and PCM@MnO_2_ NPs during 4 days incubation with Water, PBS solution plus 10% FBS, and PBS.

5. The ability of PCM@MnO_2_ NPs to consume H_2_O_2_ at different pH


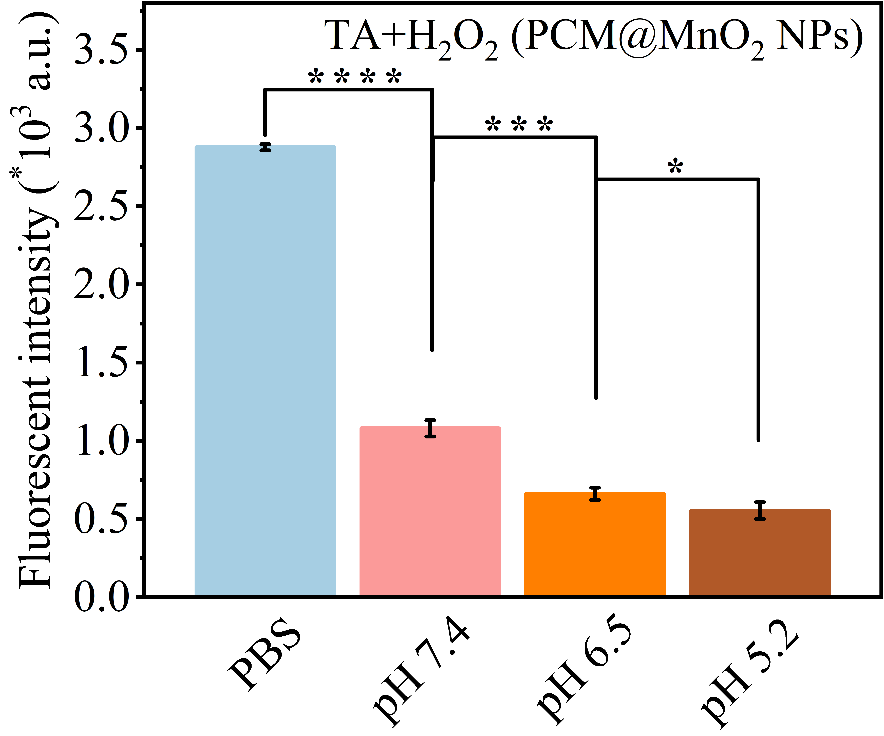


**Figure. S4** Terephthalic acid (TA) assay of PCM@MnO_2_ NPs at different pH (*n =* 3).

6. Hemolysis assay of nanoparticles


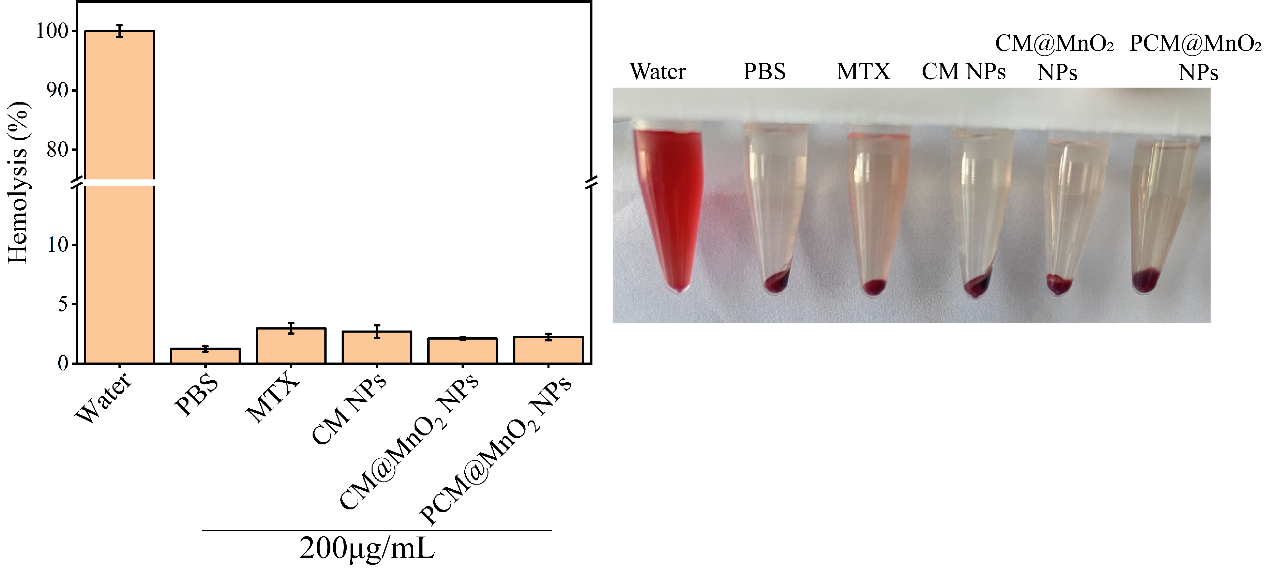


**Figure. S5** Hemolysis quantification graph and photograph of different NPs.

7. DPPH clearance of PCM@MnO_2_ NPs


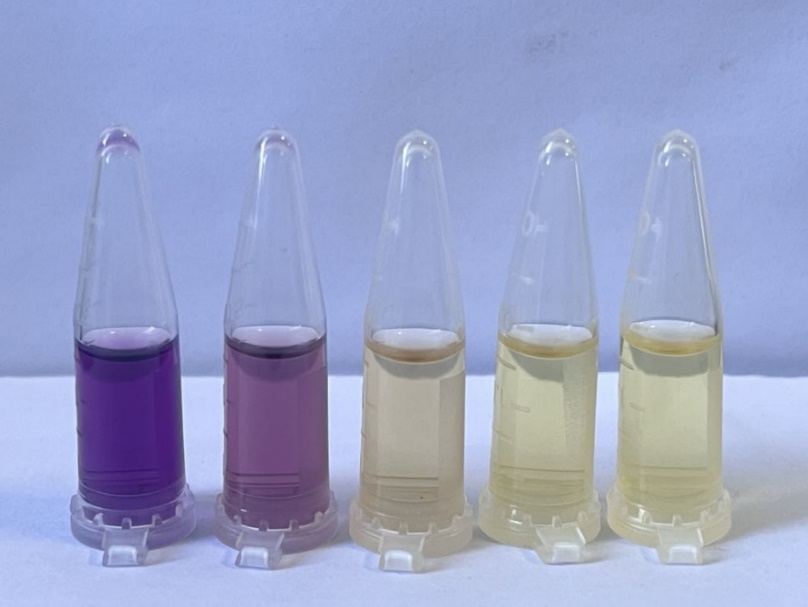


**Figure. S6** DPPH scavenging capacity of PCM@MnO_2_ NPs at different concentrations (From left to right was Control, 100, 200, and 500 μg/mL).

8. RAW264.7 cells morphology


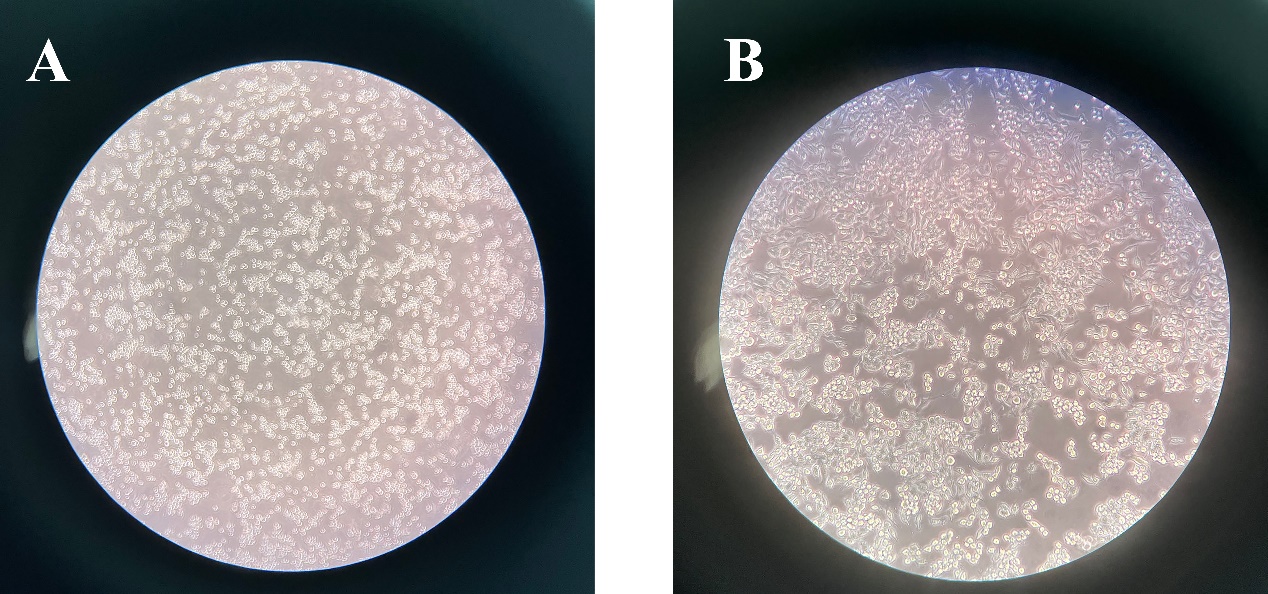


**Figure. S7** (A) and (B) represent normal and post-induced cells morphology, respectively.

9. Cells uptake


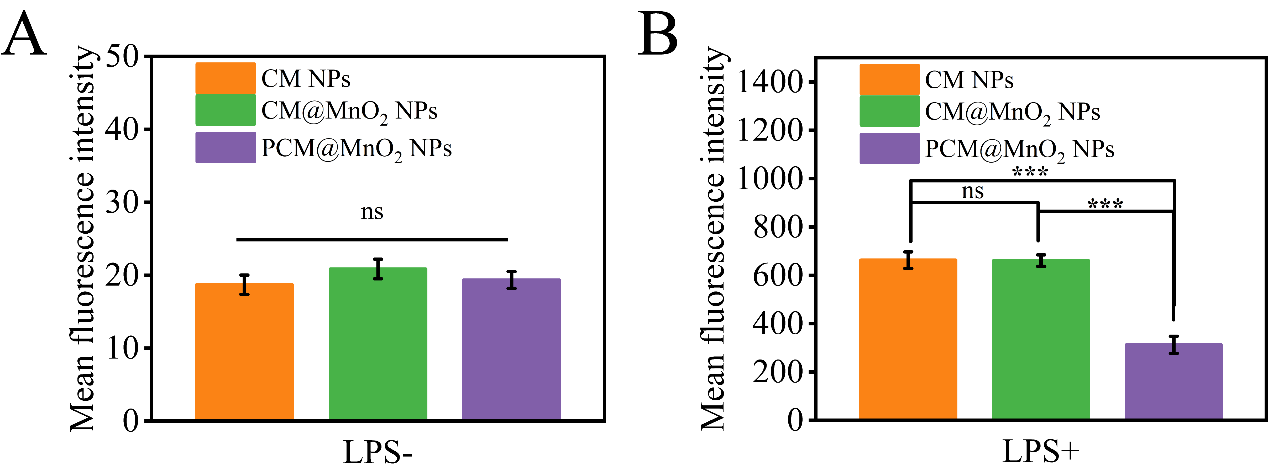


**Figure. S8** The quantitative results of the uptake by (A) normal and (B) induced macrophages

of CM NPs, CM@MnO_2_ NPs, and PCM@MnO_2_ NPs. Date represented as mean ± S.D. (*n* = 3), ****P* < 0.001. ns. represents no significant difference.

10. Inflammatory factor levels of RAW.264.7 cells


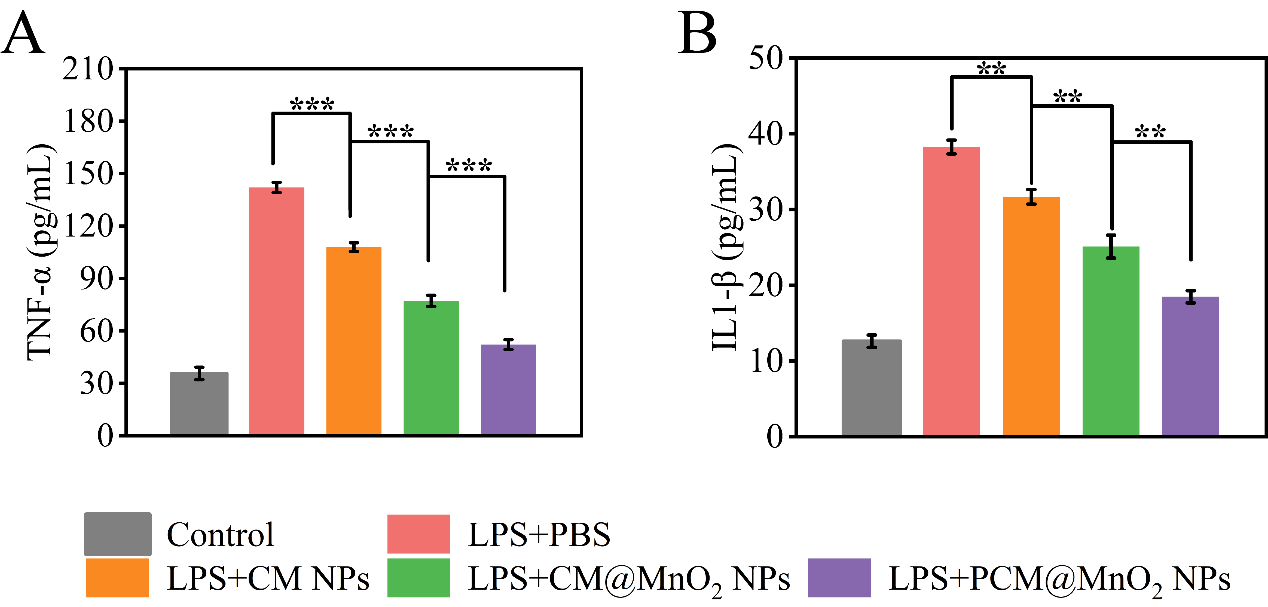


**Figure. S9** The levels of (A) TNF-α and (B) IL-1β in different groups were measured by ELISA. Date represented as mean ± S.D. (*n* = 3), ***P* < 0.01, ****P* < 0.001.

11. The results of the quantitative western blot analysis.


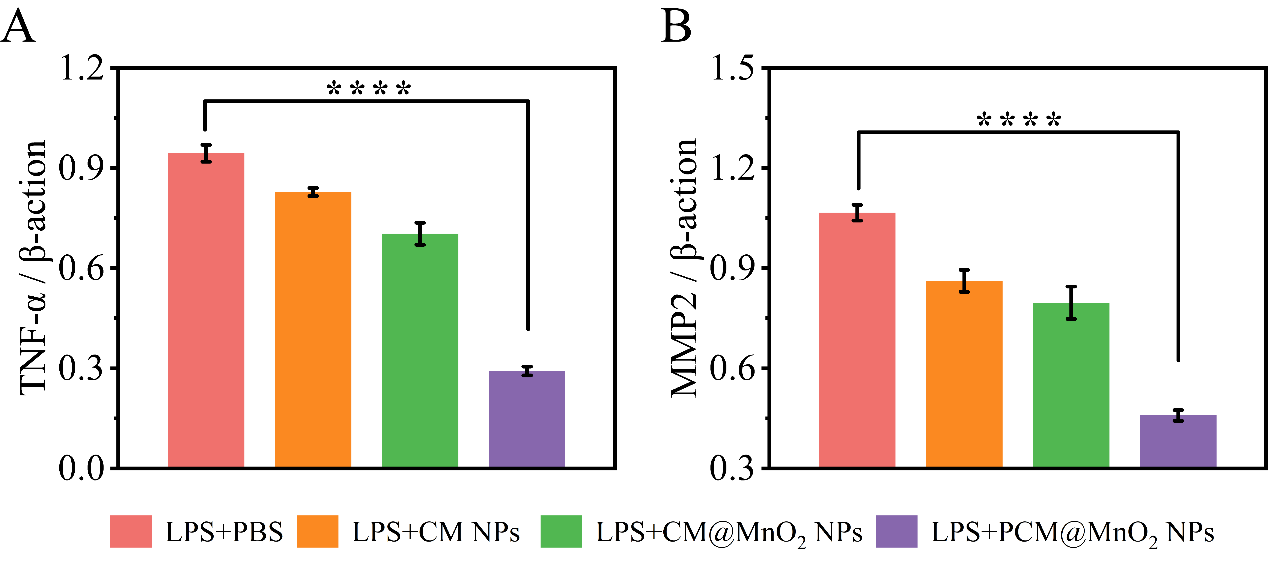


**Figure. S10.** Quantitative western blot analysis of the expression level of (A) TNF-α, and (B) MMP2 in LPS-activated RAW264.7 cells after different treatment. Date represented as mean ± S.D. (*n* = 3), *****P* < 0.0001.

12. The effect of nanoparticles on the proliferation of chondrocytes were determined by CCK-8.


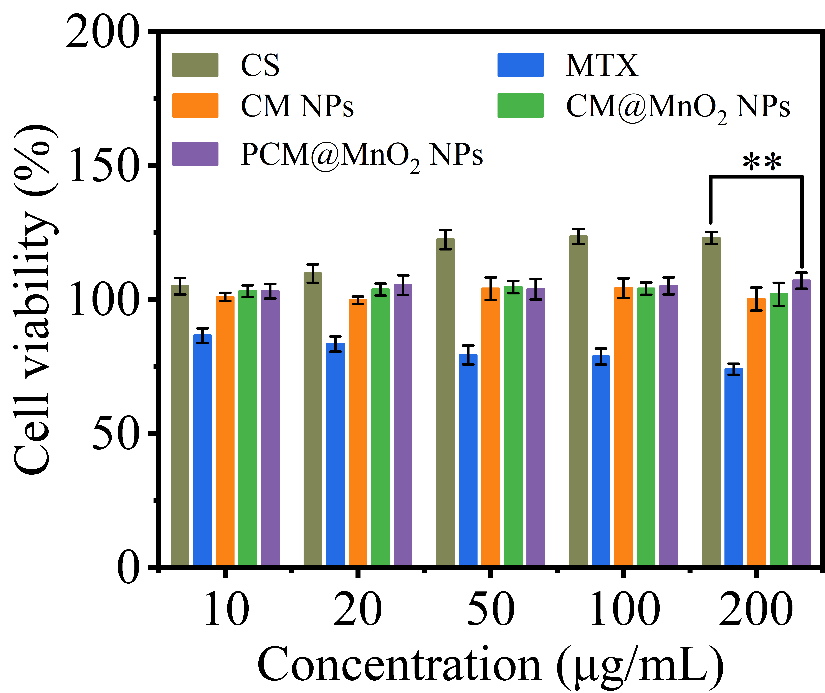


**Figure. S11.** Cell viability of IL-1β-activated chondrocytes after treatment with increasing concentrations of various formulations, as determined by CCK-8. Date represented as mean ± S.D. (*n* = 3). Statistical significance was determined by a by one-way ANOVA (*****P* < 0.0001).

13. The therapeutic effect of CS without nanoparticles.


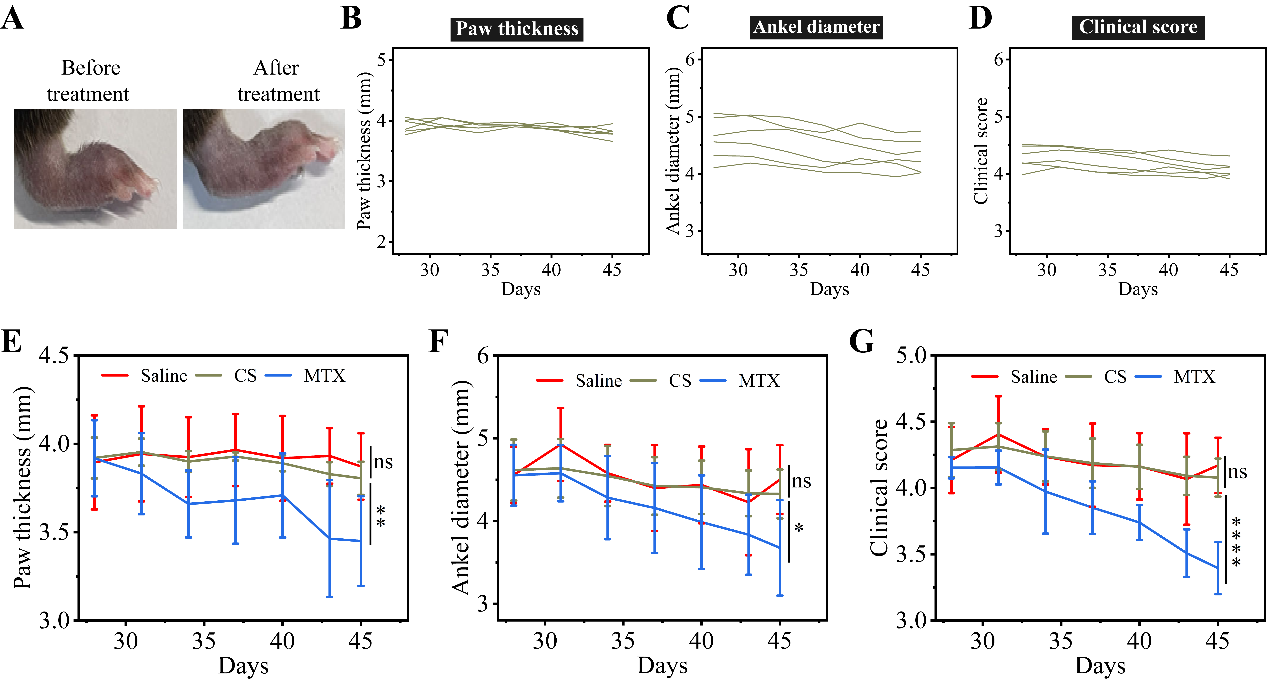


**Figure. S12** Pharmacodynamic study of CS without nanoparticles in CIA mice. (A) Comparison pictures of CIA mice treated with CS before and after treatment. (B) Changes in hind paw thickness measured during treatment for each mouse. (B) Ankle diameter of each mouse during treatment. (C) Trends in arthritis scores of each mouse during CS treatment. (E) Measurements of hind paw thickness in CIA mice, (F) changes in ankle diameter after CS treatments, and (G) evaluation of arthritis joint scores in CIA mice. The above experimental results were expressed as mean ± SD (*n* = 6), *P < 0.05, **P < 0.01, ***P < 0.001, ****P < 0.0001, ns expresses no significant difference, and analyzed by one-way ANOVA.

14. Immunofluorescence staining of macrophages


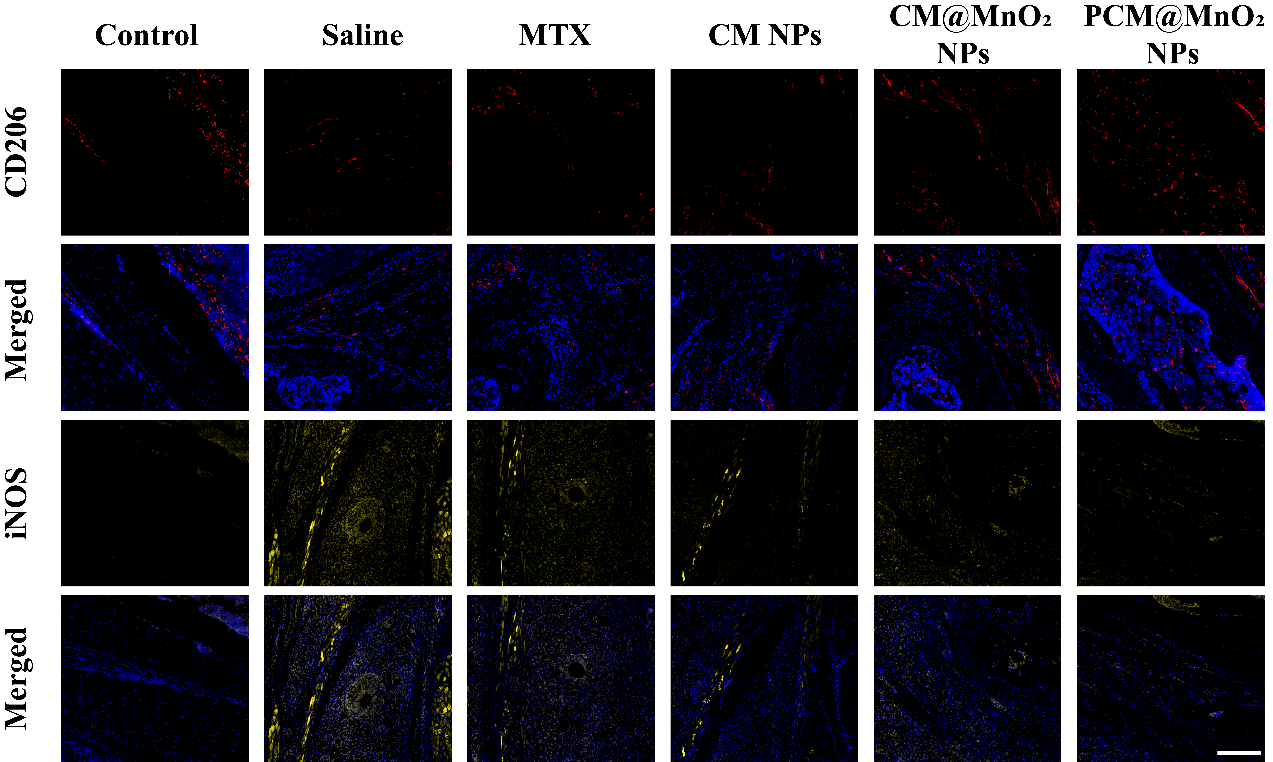


**Figure. S13** The immunofluorescence staining of iNOS and CD206 for mice ankle joints after different treatments. Scale bar *=* 200 µm.

13. The body weight of mice.


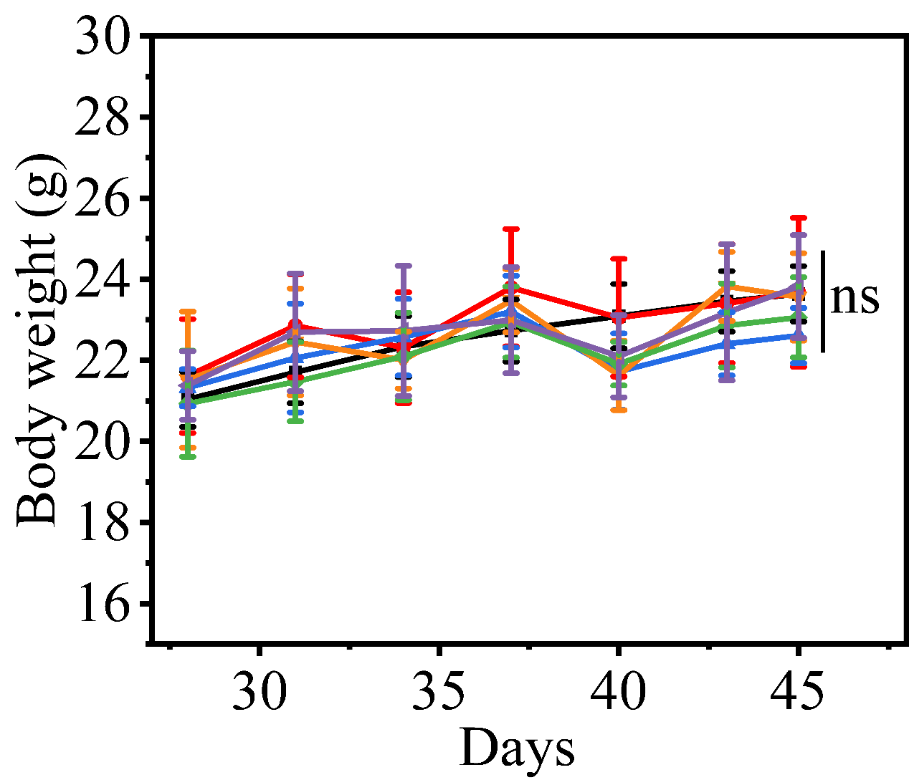


**Figure. S14** The body weight of mice in each group changed after treatment. ns. represents no significant difference.
